# Prediction and identification of *Arabidopsis thaliana *microRNAs and their mRNA targets

**DOI:** 10.1186/gb-2004-5-9-r65

**Published:** 2004-08-31

**Authors:** Xiu-Jie Wang, José L Reyes, Nam-Hai Chua, Terry Gaasterland

**Affiliations:** 1 Laboratory of Computational Genomics, The Rockefeller University, New York, NY 10021, USA; 2 Laboratory of Plant Molecular Biology, The Rockefeller University, New York, NY 10021 USA

## Abstract

**Background:**

A class of eukaryotic non-coding RNAs termed microRNAs (miRNAs) interact with target mRNAs by sequence complementarity to regulate their expression. The low abundance of some miRNAs and their time- and tissue-specific expression patterns make experimental miRNA identification difficult. We present here a computational method for genome-wide prediction of *Arabidopsis thaliana *microRNAs and their target mRNAs. This method uses characteristic features of known plant miRNAs as criteria to search for miRNAs conserved between *Arabidopsis *and *Oryza sativa*. Extensive sequence complementarity between miRNAs and their target mRNAs is used to predict miRNA-regulated *Arabidopsis *transcripts.

**Results:**

Our prediction covered 63% of known *Arabidopsis *miRNAs and identified 83 new miRNAs. Evidence for the expression of 25 predicted miRNAs came from northern blots, their presence in the *Arabidopsis *Small RNA Project database, and massively parallel signature sequencing (MPSS) data. Putative targets functionally conserved between *Arabidopsis *and *O. sativa *were identified for most newly identified miRNAs. Independent microarray data showed that the expression levels of some mRNA targets anti-correlated with the accumulation pattern of their corresponding regulatory miRNAs. The cleavage of three target mRNAs by miRNA binding was validated in 5' RACE experiments.

**Conclusions:**

We identified new plant miRNAs conserved between *Arabidopsis *and *O. sativa *and report a wide range of transcripts as potential miRNA targets. Because MPSS data are generated from polyadenylated RNA molecules, our results suggest that at least some miRNA precursors are polyadenylated at certain stages. The broad range of putative miRNA targets indicates that miRNAs participate in the regulation of a variety of biological processes.

## Abstract 2

Using bioinformatic methods, 83 novel *Arabidopsis* miRNAs have been predicted. Putative target mRNAs have been identified for most of the candidate genes.

## Background

MicroRNAs (miRNAs) are non-coding RNA molecules with important regulatory functions in eukaryotic gene expression. The majority of known mature miRNAs are about 21-23 nucleotides long and have been found in a wide range of eukaryotes, from *Arabidopsis thaliana *and *Caenorhabditis elegans *to mouse and human (reviewed in [1]). Over 300 miRNAs have been identified in different organisms to date, primarily through cloning and sequencing of short RNA molecules [2-16]. Experimental miRNA identification is technically challenging and incomplete for the following reasons: miRNAs tend to have highly constrained tissue- and time-specific expression patterns; degradation products from mRNAs and other endogenous non-coding RNAs coexist with miRNAs and are sometimes dominant in small RNA molecule samples extracted from cells. Several groups have attempted to screen for new *Arabidopsis *miRNAs by sequencing small RNA molecules, but only 19 unique *Arabidopsis *miRNAs have been found so far [12,13,15-17].

While intensive research has unmasked several aspects of miRNA function, less is known about the regulation of miRNA transcription and precursor processing. A recent study shows a 116 base-pair (bp) temporal regulatory element located approximately 1,200 bases upstream of *C. elegans let-7 *is sufficient for its specific expression at different developmental stages [18]. For some animal miRNAs, longer transcripts have been shown to exist in the nucleus before they are processed into shorter miRNA precursors [19]. Expressed sequence tag (EST) searches indicate that some human and mouse miRNAs are co-transcribed along with their upstream and downstream neighboring genes [20]. Most known animal miRNA precursors are approximately 70 nucleotides long, whereas the lengths of plant miRNA precursor vary widely, some extending up to 300 nucleotides [5,8,9,14,16]. As short mature miRNAs are generated from hairpin-structured precursors by an RNase III-like enzyme termed Dicer (reviewed in [21,22]), evidence for miRNA expression based on the presence of longer precursor RNAs is likely to be found in genome-wide expression databases.

Most known miRNAs are conserved in related species [5,8,9,14-16]. Strong sequence conservation in the mature miRNA and long hairpin structures in miRNA precursors make genome-wide computational searches for miRNAs feasible. A variety of computational methods have been applied to several animal genomes, including *Drosophila melanogaster*, *C. elegans *and humans [4,10,11,23]. In each case, a subset of computationally predicted miRNA genes was validated by northern blot hybridizations or PCR.

A known function of miRNAs is to downregulate the translation of target mRNAs through base-pairing to the target mRNA [21,24,25]. In animals, miRNAs tend to bind to the 3' untranslated region (3' UTR) of their target transcripts to repress translation. The pairing between miRNAs and their target mRNAs usually includes short bulges and/or mismatches [26-28]. In contrast, in all known cases, plant miRNAs bind to the protein-coding region of their target mRNAs with three or fewer mismatches and induce target mRNA degradation [12,15,17,29] or repress mRNA translation [30,31]. Several groups have developed computational methods to predict miRNA targets in *Arabidopsis*, *Drosophila *and humans [29,32-35].

In the work reported here, we defined and applied a computational method to predict *A. thaliana *miRNAs and their target mRNAs. Focusing on sequences that are conserved in both *A. thaliana *and *Oryza sativa *(rice), we predicted 95 *Arabidopsis *miRNAs, including 12 of 19 known miRNAs and 83 new candidates. Northern blot hybridizations specific for 18 randomly selected miRNA candidates detected the expression of 12 miRNAs. The sequences of another eight predicted miRNAs were found in the public *Arabidopsis *Small RNA Project (ASRP) database [36]. We also found massively parallel signature sequencing (MPSS) evidence for 14 known *Arabidopsis *miRNAs and 16 predicted ones. For 77 of the 83 predicted miRNAs we found putative target transcripts that were functionally conserved between *Arabidopsis *and *O. sativa*, with a signal-to-noise ratio of 4.1 to 1. Finally, we find supporting evidence for miRNA regulation of some mRNA targets using available genome-wide microarray data. The authentication of three predicted miRNA targets was validated by identification of the corresponding cleaved mRNA products.

## Results

### Prediction of *Arabidopsis *miRNAs

To predict new miRNAs by computational methods, we defined sequence and structure properties that differentiate known *Arabidopsis *miRNA sequences from random genomic sequence, and used these properties as constraints to screen intergenic regions in the *A. thaliana *genome sequences for candidate miRNAs.

Besides the well known hairpin secondary structure of miRNA precursors, the 19 unique known *Arabidopsis *miRNAs collected in Rfam [37] were evaluated for the following computable sequence properties: G+C content in mature miRNA sequences, hairpin-loop length in their precursor RNA structures, number and distribution of mismatches in the hairpin stem region containing the mature miRNA sequence, and phylogenetic conservation of mature miRNA sequences in the *O. sativa *genome. Sequences of all 19 known *Arabidopsis *miRNAs had a G+C content ranging from 38% to 70%. For 15 of the 19 miRNAs, the predicted secondary structure of their precursors, or at least one precursor if a miRNA has multiple genomic loci, had a hairpin-loop length ranging from 20 to 75 nucleotides. In the hairpin structures formed by miRNA precursors, all miRNAs were found in the stem region of the hairpin, and had at least 75% sequence complementarity to their counterparts. Fifteen of 19 miRNAs were conserved with at least 90% sequence identity in the *O. sativa *genome. Thus, constraints of G+C content between 38 and 70%, a loop length between 20 and 75 nucleotides, and a minimum of 90% sequence identity in *O. sativa *were used to predict *Arabidopsis *miRNA.

The first step was to search for potential hairpin structures in the *Arabidopsis *intergenic sequences. As most known *Arabidopsis *miRNAs are around 21 nucleotides long, we used a 21-nucleotide query window to search each intergenic region for potential miRNA precursors as follows: for each successive 21-nucleotide query subsequence, if a 21-nucleotide pairing subsequence with more than 75% sequence complementarity was found downstream within a given distance (hairpin-loop length), the entire sequence from the beginning of the query subsequence to the end of the complement pairing subsequence with a 20-nucleotide extension at each side was extracted and marked as a possible hairpin sequence (see Materials and methods for details). The minimum and maximum hairpin-loop lengths used in this prediction were 20 and 75 nucleotides. Each 21-nucleotide query subsequence and its downstream complementary subsequence were considered as 'potential 21-mer miRNA candidates' (referred to as '21-mers'). If a series of overlapping forward query sequences and their corresponding downstream pairing sequences were all identified from the same hairpin structure, each of them was initially considered as an individual 21-mer.

**Figure 1:**
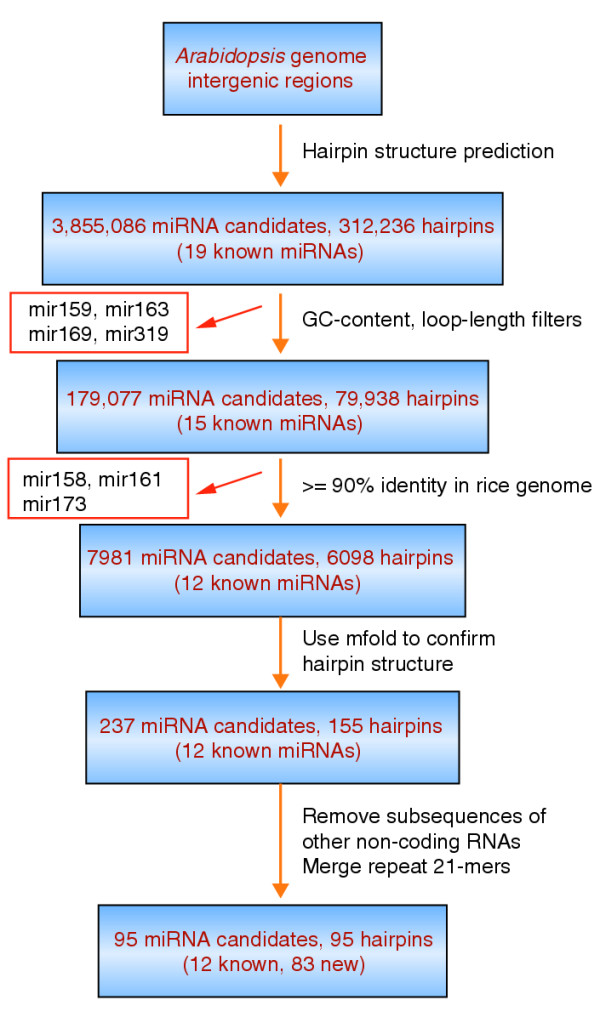
Flowchart of the *Arabidopsis *miRNA prediction procedure. The number of predicted miRNA candidates and potential miRNA precursors (hairpins) is shown in blue bars. The number of known *Arabidopsis *miRNAs included in each prediction step is shown in parentheses. Known *Arabidopsis *miRNAs rejected by each prediction step are shown in red boxes.

The second step was to parse miRNA candidates according to their nucleotide composition and sequence conservation. A filter of G+C content between 38 and 70% was applied to all 21-mers obtained from the above step, followed by a requirement for more than 90% sequence identity in the *O. sativa *genome. The secondary structures of the resulting candidates were evaluated by mfold [38]. Only 21-mers whose *Arabidopsis *precursor and corresponding rice ortholog precursor both had putative stem-loop structures as their lowest free energy form reported by mfold were retained. Because some non-coding RNA genes were not included in the current *Arabidopsis *gene annotation, orthologs of known non-coding RNA genes other than miRNAs were subsequently removed by aligning the 21-mers to non-coding RNAs collected in Rfam with BLASTN (version 2.2.6) [37]. The 21-mers that passed all sequence and structure filters above were considered as final miRNA candidates. A summary of the prediction algorithm is shown in Figure 1.

In cases where two or more overlapping 21-mer miRNA candidates from the same precursor were collected in the final miRNA candidate set, each miRNA candidate was scored using the following formula:

miRNA_score _= number of mismatches + (2 × number of nucleotides in terminal mismatches) + (number of nucleotides in internal bulges/number of internal bulges) + 1 if the miRNA sequence does not start with U.

The term 'terminal mismatches' in the formula above refers to consecutive mismatches among the beginning and/or ending nucleotides of a mature miRNA sequence. The term 'bulge' refers to a series of mismatched nucleotides. Because the sequences of most known miRNAs start with a U, a U-start preference was used in the formula above by penalizing non-U-start sequences. The sequence with the lowest miRNA_score _from a series of overlapping 21-mers was selected as the final miRNA candidate.

In total, we predicted 95 miRNA candidates in the *Arabidopsis *genome, including 12 known *Arabidopsis *miRNAs and 83 new candidates. The former group corresponds to 63% of known *Arabidopsis *miRNAs to date (12 of 19). The remaining seven known miRNAs not included in the current prediction were filtered out as a result of their lower sequence conservation in the rice genome or longer loop length in their secondary structure, as outlined in Figure 1. Because of the complementarity between the two DNA strands of a given genome region, theoretically there should be two sequence possibilities for a predicted miRNA: the predicted sequence itself or, alternatively, its reverse complementary sequence located on the opposite strand of the genome. In many cases, however, owing to G::U pairing in RNA structure prediction, the complementary sequence of a miRNA precursor did not always exhibit a hairpin structure as its lowest energy folding form because the complement of a G::U pair, that is, C::A, altered the secondary structure. Thus, we were able to identify the coding strand of most predicted miRNA candidates through secondary structure evaluation. Furthermore, as described in the following sections, the sequences/partial sequences of some miRNA candidates or their precursors could be found in the *Arabidopsis *MPSS data used. As most MPSS data probably represent the expression of their associated miRNAs, we were able to use them to predict the miRNA coding strand. The coding strand of miRNA candidates that were contained in the ASRP database was determined according to cloned RNA sequences (see below for details). The complete list of predicted miRNAs is shown in Additional data file 1.

### Experimental validation of predicted miRNAs

**Figure 2:**
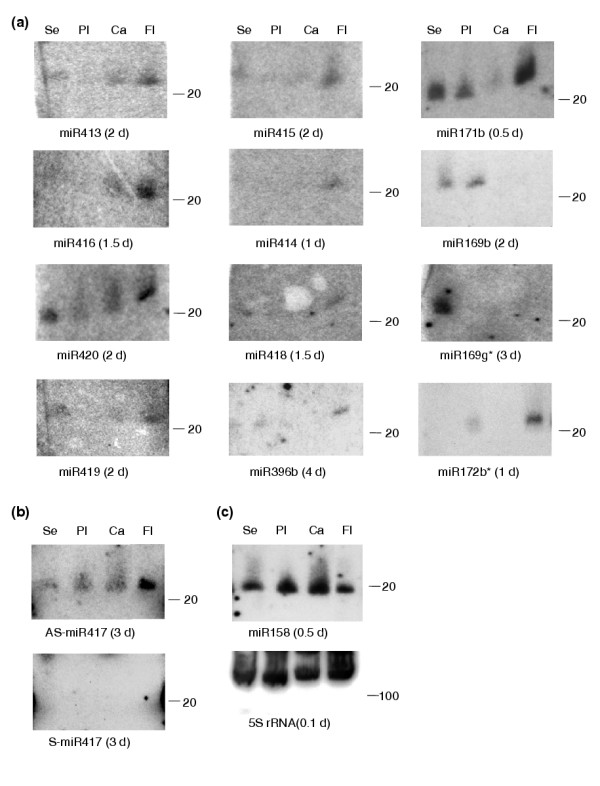
Northern blot analysis of predicted miRNAs. Total RNA (20 μg) from 2-day-old seedlings (Se), 4-week-old adult plants (Pl), root-regenerated calluses (Ca), and mixed-stage flowers (Fl) was resolved in a 15% polyacrylamide/8 M urea gel for northern blot analysis. **(a) **Hybridization signal from confirmed miRNAs. **(b) **Antisense and sense oligonucleotides (indicated by AS and S, respectively) were used to confirm the polarity of miR417. **(c) **Hybridization signal for miR158 and 5S rRNA as indicated. The number next to each panel represents the position of RNA markers in nucleotides. In all cases the number in parentheses indicates the time of film exposure in days.

**Figure 3:**
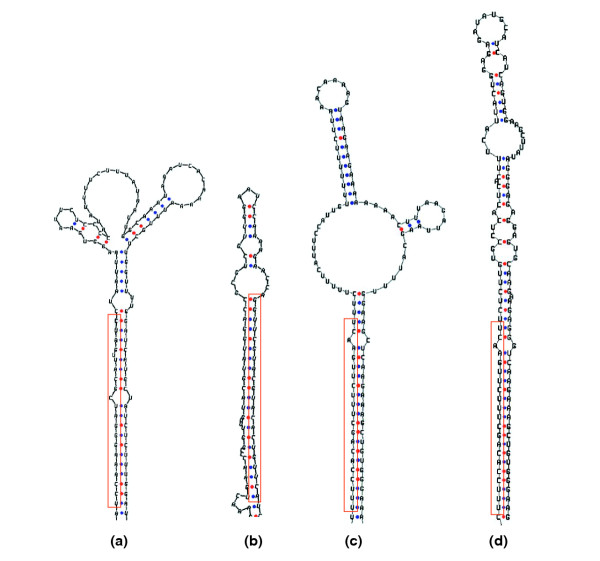
Putative secondary structures of selected miRNA precursors. **(a-c) **Secondary structures of predicted precursors of *Arabidopsis *miR393a, miR416 and miR396b, respectively. **(d) **pri-mir structure of proposed *O. sativa *homolog of *Arabidopsis *miR396b shown in (**c)**. Sequences of mature miRNAs are marked with a red box.

To gain support for the expression of the predicted miRNAs, northern blot hybridizations were carried out using RNA samples from different tissues selected to cover a spectrum of potential miRNA expression patterns. Using strand-specific oligonucleotide probes, positive signals of expression were detected for 14 out of 18 miRNA candidates tested. The results for all newly identified miRNAs are shown in Figure 2a and 2b. Oligonucleotide probes against the antisense strand of different miRNA candidates were used as negative controls, and none produced any signal, as shown for miR417 in Figure 2b. Note that an extended exposure time was needed to detect expression of most miRNAs (indicated by a number in days in parentheses in Figure 2), suggesting that their abundance is significantly lower than that of other known miRNAs (that is, miR158 and miR159a in Figure 2c, and data not shown). In this analysis we also included 10 21-mers that were rejected by our miRNA prediction criteria as negative controls to evaluate the specificity of northern blot hybridization; as expected none of them produced a positive signal. The secondary structures of a few selected northern blot hybridization-positive miRNA candidates are shown in Figure 3. A full list of the secondary structures of predicted precursors of *Arabidopsis *miRNA candidates and their rice orthologs is available in Additional data file 2.

Among the 14 miRNAs that produced positive signals in the northern blot hybridizations, two are close paralogs of known miRNAs; miR169b is a paralog of miR169 and miR171b is a paralog of miR170. Because it is impossible to distinguish closely related sequences by northern blot hybridization, we were unable to rule out the possibility that signals detected by probes for miR169b and miR171b were contributed by their known miRNA paralogs. However, as miR169b was also identified in the ASRP database (see next section), we were able to conclude that miR169b was a real miRNA. Thus, 12 candidates validated by northern blot hybridization should be annotated as *bona fide *miRNAs (see Table 1 for a summary).

### Cloning evidence for predicted miRNAs

**Figure 4:**
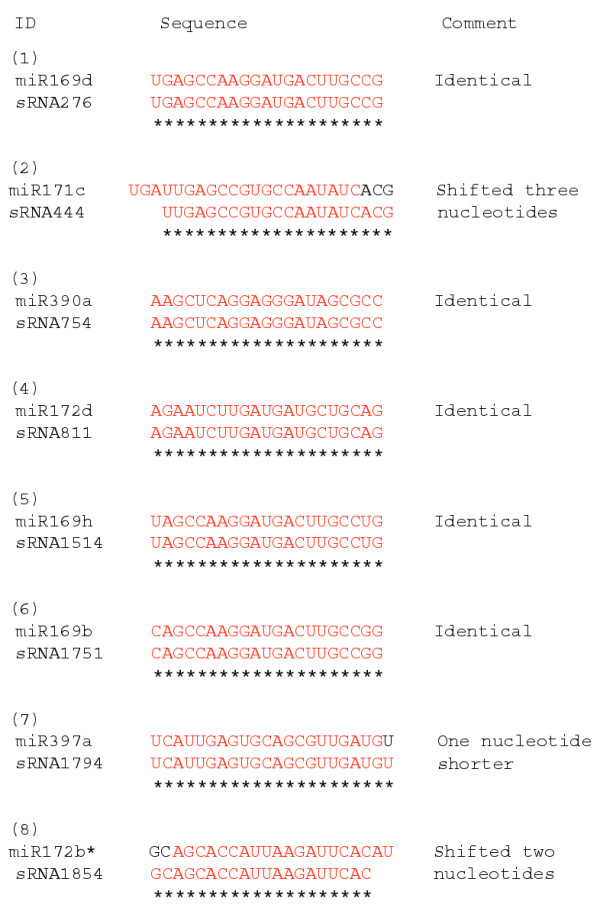
Comparison of predicted miRNAs with sequences in the *Arabidopsis *ASRP database. Sequences from the ASRP database are named as 'sRNA' followed by clone numbers. Sequences of predicted miRNAs and sequences from ASRP database are shown in red; miRNA sequences extended according to cloned RNA sequences are in black. The final miRNA sequences reported in Additional data file 1 are marked with asterisks.

An ASRP database has recently been made publicly available [36]. Sequences in the ASRP database were collected by cloning small RNA molecules with similar size to miRNAs and siRNAs [39]. To check whether any of our predicted miRNAs can be identified by a standard RNA cloning method, we compared the 83 predicted miRNA candidates with all sequences in the ASRP database. Eight newly predicted miRNA candidates were found in the ASRP database (Figure 4). Among them, five were identical to one or more cloned RNA molecules, indicating that we had correctly predicted the 5' and 3' ends and the actual length of these miRNA candidates. For the other three candidates, our predicted sequences were either shorter than, or a few nucleotides shifted from, their corresponding clones in the ASRP database. The exact sequences of these three miRNA candidates were then corrected according to the corresponding sequences in the ASRP database. The expression of miR169b and miR172b* was also detected by northern blot hybridization (Figure 2a). Although miR169h was present in the ASRP database, it could not be detected by northern blot hybridization (see Additional data file 1). According to the current miRNA annotation criteria [22], these eight predicted miRNA candidates with corresponding cloned sequences in the ASRP database should be annotated as *bona fide *miRNAs.

### MPSS evidence for known and predicted *Arabidopsis *miRNAs

To further validate the predicted miRNA molecules, we took advantage of available *Arabidopsis *massively parallel signature sequencing (MPSS) data. The MPSS sequencing technology identifies unique 17-nucleotide sequences present in cDNA molecules originated from polyadenylated RNA extracted from a cell sample. By inserting cDNA molecules into a cloning vector containing distinct 32-mer oligonucleotide tags, the MPSS technology ensures that each cDNA molecule is ligated to a unique tag and that more than 99% of the total cDNAs are represented after the cloning step. Tagged cDNAs are then amplified by PCR and hybridized to microbeads that have been precoated with multiple copies of unique anti-tags complementary to one type of 32-nucleotide tag. The expression level of a particular transcript is measured by counting the number of distinct microbeads that contain the same 17-nucleotide cDNA sequence. The MPSS technology does not require prior knowledge of a gene's sequence and thus can identify novel or rarely expressed genes. For a complete description, see [40,41].

To assess the degree to which MPSS data could be used to support predicted miRNAs, we inspected the 19 known *Arabidopsis *miRNAs for unique representation in public *Arabidopsis *MPSS datasets and in our own MPSS datasets derived from a variety of tissues and conditions (see Materials and methods for details) [42-44]. We compared the intergenic genomic sequence flanking the 19 known *Arabidopsis *miRNAs with the MPSS data. We found 30 MPSS signature sequences that were identical to subsequences within the flanking 500-bp sequences either upstream or downstream of 14 known miRNAs (see Additional data file 3). All 30 MPSS sequences were reported in both the public and private MPSS datasets. They occurred upstream, downstream or partially overlapping with known mature miRNAs. Despite the highly repetitive nature of the *Arabidopsis *genome, 28 of the 30 MPSS signatures mapped uniquely to only one miRNA locus, with no matches elsewhere in the genome. Two genomic loci were found for each of the two exceptional MPSS signatures MPSS78528 and MPSS28409. For MPSS78528, the associated miRNA mir162 appeared twice in the *Arabidopsis *genome (upstream of At5g08180 and upstream of At5g23060) and the MPSS sequence mapped exactly to those regions. For MPSS28409, its second genomic match was on the opposite strand of an intron in gene At3g04740, which was very unlikely to be a source for MPSS sequences because samples for MPSS were prepared from mRNA or other type of polyadenylated RNA molecules, in which introns should have been processed. Thus, the MPSS data accurately reflected the expression of 14 known *Arabidopsis *miRNAs from a total of 19, indicating that it can be used as a source of indirect experimental support for the expression of predicted miRNAs.

We then assessed the presence of MPSS signature sequences for the 83 predicted miRNAs. Using the approach described above, 23 MPSS signature sequences corresponding to the flanking sequences of 16 predicted miRNAs were found (see Additional data file 1). All 23 MPSS signature sequences were present in both the public and our own MPSS datasets, and mapped uniquely to the miRNA flanking sequence. The expression of nine miRNA candidates supported by MPSS data was also tested by northern blot hybridization, with eight of them producing a positive signal. Another three miRNAs with MPSS data were found in the ASRP database (see previous section and Additional data file 1). These results indicate that MPSS data indeed represent the expression of predicted miRNAs.

### Comparison of predicted miRNAs to known *Arabidopsis *miRNAs

**Figure 5:**
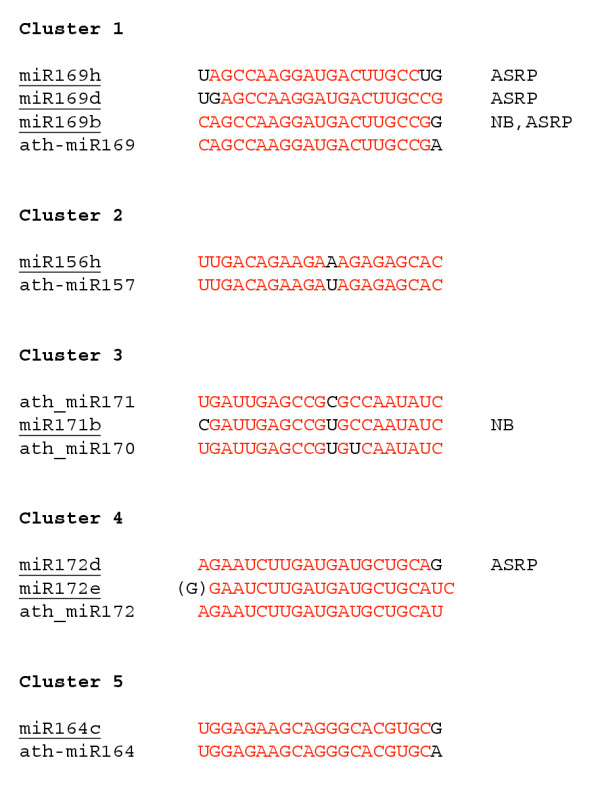
Clusters of predicted miRNAs with known *Arabidopsis *miRNAs. Identical nucleotides in predicted (underlined names) and known *Arabidopsis *miRNAs are highlighted in red; differences are highlighted in black; adjacent genomic sequences are shown in black in parentheses. NB indicates miRNAs whose expression was detected as positive by northern blot hybridization; ASRP indicates sequences present in the ASRP database.

To explore the relationship of predicted miRNAs to known *Arabidopsis *miRNAs, we compared the sequences of all 83 miRNA candidates from our prediction with sequences of the 19 known *Arabidopsis *miRNAs. Eight predicted *Arabidopsis *miRNAs exhibited high sequence similarity to one or more known *Arabidopsis *miRNAs and could be grouped into five clusters (Figure 5). We could not find convincing evidence that *Arabidopsis *and animal miRNAs are related, as clustering of these required the insertion of multiple gaps in the alignments (data not shown).

### Putative mRNA targets of predicted *Arabidopsis *miRNAs

A previous study has predicted that most known plant miRNAs bind to the protein-coding region of their mRNA target with nearly perfect sequence complementarity, and degrade the target mRNA in a way similar to RNA interference (RNAi) [29]. Analysis of several targets has now confirmed this prediction, making it feasible to identify plant miRNA targets [12,15,16]. We developed a computational method based on the Smith-Waterman nucleotide-alignment algorithm to predict mRNA targets for the 83 newly identified miRNA candidates reported in this paper (see Materials and methods for details). Focusing on miRNA complementary sites that were conserved in both *Arabidopsis *and *O. sativa*, our method was able to identify 94% of previously confirmed or predicted mRNA targets for known conserved *Arabidopsis *miRNAs. Applying the method to the 83 predicted *Arabidopsis *miRNA candidates and their *O. sativa *orthologs, we predicted 371 conserved mRNA targets for 77 predicted *Arabidopsis *miRNAs, with an average of 4.8 targets per miRNA. The signal-to-noise ratio of the miRNA targets prediction was 4.1:1 when using randomly permuted sequences with the same nucleotide composition to miRNA sequences as negative controls that went through the same target prediction process. A complete list of these predicted target mRNAs and their pairings with miRNA sequences is available in Additional data file 4.

Of the 371 predicted miRNA targets, 10 were potential targets of two independent miRNAs, one (At3g54460 mRNA) was a potential target of three different miRNAs (At1g60020_5_14, At3g27883_1009, At5g62160_613_rc), and the rest were targets of a single miRNA. We assessed the biological functions of all predicted miRNA targets using gene ontology (GO) [45]. GO terms for 254 targets were found in the molecular function class. Molecular functions of the putative miRNA targets included transcription regulator activity, catalytic activity, nucleic acid binding, and so on, as summarized in Table 2. As some proteins were classified in more than one molecular function category, the total number of targets listed in different function categories in Table 2 exceeds the number of targets with GO function assignment.

Consistent with previous reports [29], a large proportion of predicted targets encoded proteins with transcription regulatory activity, corresponding to 50% of total targets with GO annotation (129/254). One interesting phenomenon was that most transcription regulators in the miRNA target set were plant specific, such as MYB, AP2, NAC, GRAS, SBP and WRKY family transcription factors (Table 3). For example, the miRNA target set included 10 plant specific NAC-domain-containing transcription factors, corresponding to 9% of total NAC-domain-containing transcription factors encoded by the *A. thaliana *genome. In contrast, 139 genes encoding a general transcription factor bHLH were found in the *A. thaliana *genome, but only three were putative miRNA targets.

We analyzed the expression patterns of potential targets to look for indications that they were under miRNA regulation. Twelve of the 14 miRNAs confirmed by northern blot hybridization showed an increased accumulation in flower tissue compared to the other tissues tested (Figure 2), suggesting a role for miRNAs in regulating flower-specific events. In a search of *Arabidopsis *microarray gene expression data available from The *Arabidopsis *Information Resource (TAIR) [46], we found the expression profile for 11 predicted mRNA targets that can base-pair nearly perfectly with five confirmed flower-abundant miRNAs. We hypothesized that expression levels of these targets in flower tissue could be decreased as compared to whole plant RNA samples as a result of mRNA cleavage induced by miRNA regulation. Accordingly, a reduced expression level (more than 1.25-fold decrement) was found for eight genes in total flower mRNA compared to total whole plant mRNA, with another three whose expression was almost unchanged (Table 4). A *t*-test on the possibility of decreased expression between transcripts listed in Table 4 and in the entire microarray data resulted in a *p*-value of 0.04, indicating that the decreased expression observed for predicted miRNA targets is significantly different from the general expression pattern of the entire microarray data.

**Figure 6:**
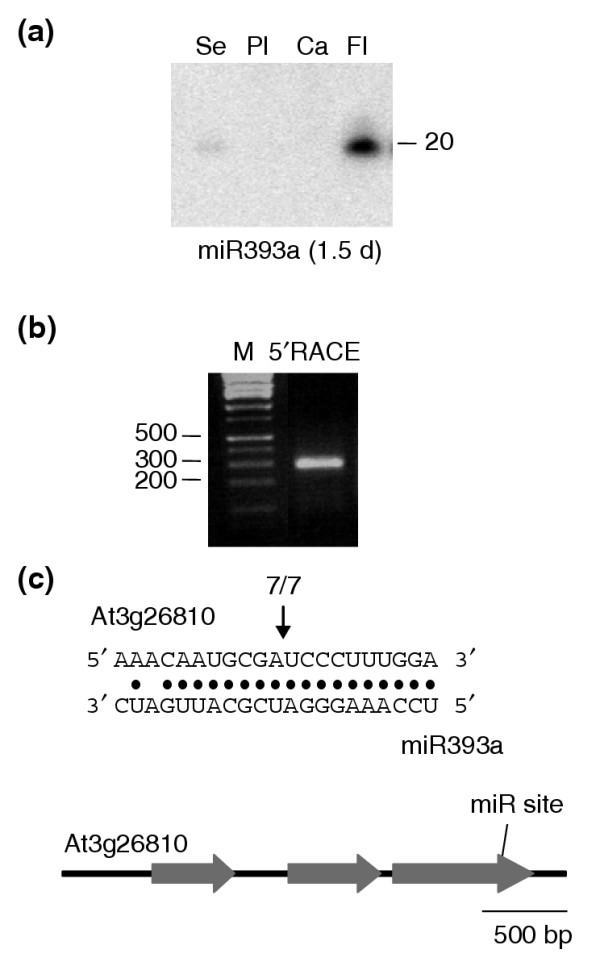
Validation of a miRNA-cleaved mRNA target. **(a) **Northern blot analysis of miR393a showing its expression pattern. Samples are identical to those in Figure 2b. **(b) **The 5' RACE product for the predicted target gene At3g26810 amplified by PCR is shown in the ethidium bromide-stained agarose gel. **(c) **The 5' end of the cleaved product determined by sequencing is indicated by an arrow in the miRNA:mRNA base-pairing diagram, along with the number of clones analyzed. The gene structure of At3g26810 and location of the miRNA-binding site are shown at the bottom.

Target mRNA fragments resulting from miRNA-guided cleavage are characterized by having a 5' phosphate group, and cleavage occurs near the middle of the base-pairing interaction region with the miRNA molecule. Using a modified RNA ligase-mediated 5' rapid amplification of cDNA ends (5' RACE) protocol, we were able to detect and clone the At3g26810 mRNA fragment corresponding precisely to the predicted product of miRNA processing (Figure 6). Two other genes, At3g62980 (TIR1) and At1g12820, share extensive sequence homology with At3g26810 and were also predicted to be targets of miR393a. Consistent with this, we also identified the corresponding RNA fragments derived from miRNA cleavage by 5' RACE (data not shown). We were not able to identify other targets from flower RNA samples using a similar approach. The microarray data used in this tissue comparison experiment includes around 7,400 genes only (about a quarter of the entire *Arabidopsis *genome). Thus, we expect the expression profile of more mRNA targets to be determined as more whole-genome tissue comparison data is available.

## Discussion

We have developed and applied a computational method to predict 95 *Arabidopsis *miRNAs, which include 12 known ones and 83 new sequences. All 83 new miRNAs are conserved with more than 90% identity across the *Arabidopsis *and rice genomes. The expression of 19 new miRNAs was confirmed by northern blot hybridization or found in a publicly available database of small RNA sequences. MPSS data support was also found for 14 known and 16 predicted *Arabidopsis *miRNAs. Of the 16 miRNAs, 10 were confirmed by northern blot hybridization or by their presence in the ASRP database, and six have MPSS data only. In total, we have found direct or indirect experimental evidence for 25 predicted miRNAs. We expect more evidence to be found for other predicted miRNAs as independent experimental data, such as small RNA sequencing and MPSS data, grow. Among the 83 predicted miRNAs, eight have strong sequence similarity with known plant miRNAs. The prediction results and supporting experimental evidence are summarized in Table 5. Additional data file 1 summarizes the corresponding evidence for known miRNAs and contains additional detailed information for each new candidate. Potential functionally conserved mRNA targets were found for 77 predicted miRNAs.

### Assessment of miRNA prediction

The prediction method developed in this study uses computable sequence and structure properties that characterize the majority of the known *Arabidopsis *miRNA genes to constrain the miRNA search space. Parameters used in the prediction were selected to minimize false positives while maximizing true positives. Thus, seven known miRNAs (37%) were missed using our selected parameters. However, relaxing the loop length range to include all known miRNAs increased the number of candidate hairpins from around 180,000 to around 337,000 (a 53% increase). As the method requires stringent miRNA sequence conservation between *Arabidopsis *and *O. sativa*, miRNAs with little or no sequence conservation in other genomes will be overlooked by this method. Given the current knowledge of miRNAs, it is difficult to develop computational methods to distinguish non-conserved miRNAs from noise. The prediction method developed here is not specific for *Arabidopsis *and can be applied to other pairs of related genomes as well.

We attained a 67% success rate of northern blot hybridization on all tested miRNA candidates, demonstrating the expression of 12 miRNAs from a total of 18 tested candidates. Failure to detect miRNA candidates by northern blot hybridization could be due to the limited number of sample tissues tested, as specific miRNAs may be expressed only under particular conditions (stimuli and/or developmental stages) or in specific cell types. For instance, further analysis of miR169g* (shown in Figure 2a) indicated a higher accumulation in mature siliques than in the seedling stage (J.L.R. and N-H.C., unpublished work). This can only be determined by detailed study of individual miRNAs, which is not possible in a general screening such as the one presented here. Alternatively, the expression level of certain miRNAs may fall below the detection limit of our assay conditions. Consistent with this idea, in all cases the confirmed miRNAs were detected only after an extended period of autoradiography (2-3 days), as compared with known miRNAs that were more easily detected (see Figure 2 and data not shown). The low abundance of the newly identified miRNAs is in agreement with the limited number of miRNAs identified to date using the established cloning strategies.

### MPSS support for miRNAs and possible polyadenylation of precursor transcripts

We made an intriguing discovery by identifying known *Arabidopsis *miRNAs and their approximate precursor sequences in MPSS polyadenylated transcript datasets. All but two MPSS sequences reported here uniquely map to the mature miRNA sequence or to the flanking sequence 500 nucleotides upstream or downstream (see Results). The MPSS sequence locations and orientations indicate that they are not transcripts derived from surrounding annotated genes. All but one tested miRNA candidates with MPSS evidence produced positive signals in northern blot hybridizations. As MPSS cDNA libraries are generated using polyadenylated RNA molecules [40,41,43,44], the presence of 14 known *Arabidopsis *miRNAs from a total of 19 in these datasets strongly indicates that at least some, if not all, plant miRNAs have a polyadenylated precursor form at some stage of their biogenesis.

Predicted miRNAs detected by both northern blot hybridization and MPSS have consistent tissue-specific expression profiles under both methods. This supports the notion that the MPSS data reflect miRNA expression patterns. The public MPSS datasets are accessible only through an online interface that allows direct query of 17-nucleotide MPSS sequences. Direct comparison of the public sequences and predicted miRNAs was not possible. Thus, we were limited in our analysis to inquire whether a private MPSS sequence was also in the public MPSS dataset. Consequently, only MPSS sequences that appeared in the private set alone or in both sets were available to support miRNA prediction. The public MPSS dataset has 120-fold more signature sequences (more than 12 million additional tags) than our private MPSS dataset (approximately 94,000 tags total). Thus, we expect far more MPSS evidence for expression of the predicted miRNAs to be found in the public MPSS datasets when the public MPSS data are available to be compared locally.

### Target mRNAs for predicted miRNAs

We used phylogenetic conservation as a constraint on miRNA target mRNA prediction: only transcripts that had at least one *O. sativa *functional ortholog among the top 500 rice miRNA targets were considered as potential miRNA target genes. Because all miRNA candidates reported here are highly conserved in rice, it is expected that their mRNA targets should also be conserved. Transcripts encoding proteins with ambiguous annotations, such as those for hypothetical proteins, expressed proteins and putative proteins, are not included in the target prediction because of the difficulty in identifying their orthologs in the *O. sativa *genome. Thus, the absence of predicted mRNA targets for the minority of the miRNAs may be due to the unfinished annotation of the *O. sativa *genome or to the divergence of target mRNA sequences that may preclude its identification.

Gaps and mismatches are commonly seen in known animal miRNA::mRNA base-pairing interactions and, as a result, miRNA binding represses the translation of their targets [1]. Although in most known cases plant miRNAs tend to pair nearly perfectly with their target mRNAs and induce mRNA cleavage [12,15,17,29], recent evidence has shown that plant miRNAs can also repress target mRNA translation in a way similar to that of animal miRNAs [1,30,31]. To further explore the function of *Arabidopsis *miRNAs in target mRNA translation repression, in this prediction we allowed gaps and mismatches in the putative *Arabidopsis *miRNA::mRNA pairs. The free energy of 90% of the predicted miRNA::mRNA pairs is lower than the average free energy of known animal miRNA::mRNA pairs (ΔG = -14 kcal/mol) [32], indicating that the predicted miRNA::mRNA pairs are potentially stable at the energy level if similar interactions are present in plants (see Additional data file 4).

Our prediction of target mRNAs for the new 83 miRNA candidates reveals that a broad functional range of genes may be regulated by miRNAs (Table 4 and Additional data file 4). As in previous findings, the predicted miRNA targets were enriched for transcription factors [29]. Our prediction also included transcripts encoding proteins with transcription regulator activity, catalytic activity, signal transducer activity and translation regulator activity. This result is consistent with recent findings in animal miRNA targets, and suggests a broader role for miRNA regulation in plant gene expression [29,32-35].

We took advantage of available microarray data to assess the relative expression levels of potential mRNA targets in tissues in which their miRNAs were expressed. For mRNA targets of several newly identified miRNAs, we found reduced expression levels in RNA samples from flowers compared to the whole plant. Accordingly, we identified the cleavage product of At3g26810 mRNA, and those of another two homologous genes, At1g12820 and TIR1 (At3g62980), to confirm them as targets of the same miRNA (miR393a). These genes encode F-box proteins, and TIR1 in particular is involved in auxin-mediated protein degradation [47]. Interestingly, F-box proteins are another group over-represented among the target mRNAs (48 out of 254, or 19%), while there are around 700 F-box proteins encoded in the *Arabidopsis *genome (2.1%) [48]. Remarkably, these are the first confirmed plant miRNA targets that are not transcription factors, with the exception of DCL1 and AGO1. The identity and expression pattern of a target mRNA can help identify the specific expression profile of its corresponding miRNA. Tissues with low mRNA expression levels should be checked carefully for miRNA expression. Currently, this kind of search is limited by the availability of genome-wide and tissue-/time-specific microarray data. As such data accumulate, their analysis will enrich our understanding of the different biological processes regulated by microRNAs.

## Materials and methods

### Computational prediction of *Arabidopsis *miRNAs

The *Arabidopsis *genome version 3 and the *O. sativa *genome released by The Institute for Genome Research (TIGR) on 22 July 2002 [49] and 24 January 2003 [50], respectively, were used for the present study. Intergenic regions of both the *A. thaliana *and the *O. sativa *genomes were extracted according to the annotations provided by TIGR. A scanning algorithm implemented in the Perl programming language was used to search for possible hairpin structures within each intergenic sequence in *A. thaliana*. The method inspects each successive 21-nucleotide query window in each intergenic region, with two nucleotide increments, and searches for downstream complementary sequences with up to 25% mismatches. We restricted the distance from the last base of the forward query sequence and the first base of the downstream complementary pairing sequence to a minimum of 10 nucleotides and a maximum of 150 nucleotides (loop length). For each 21-nucleotide query, the loop length was increased one nucleotide at a time, and all downstream 21-nucleotide pairing sequences with more than 75% identity to the complement of the query sequence were considered as possible 'pairing sequences'. For each query sequence, the downstream complementary sequence with the fewest mismatches was saved as the pair sequence for the query. Insertions and deletions were allowed in the alignment and were counted as mismatches. Sequences from the start of a qualified querying sequence to the end of its downstream complementary pairing sequence were considered as a potential miRNA precursor with putative hairpin structure. Twenty extra nucleotides were extracted from the genome at each end of a potential miRNA precursor for the purpose of structure check using mfold [38].

A G+C-content filter and a loop-length filter were applied to the 312,236 hairpin structures obtained. Only hairpins with a loop length between 20 and 75 nucleotides and 21-mer sequences with a G+C-content between 38 and 70% were analyzed further. The remaining 21-mer sequence pairs were aligned with rice intergenic regions using BLASTN (version 2.2.6) to identify homologous sequences in *O. sativa *intergenic regions with 90% or higher sequence identity. The secondary structure of *Arabidopsis *miRNA candidate precursors and their rice precursor orthologs was evaluated using mfold [38]. Only 21-mers whose *Arabidopsis *precursor and rice ortholog precursor both had a hairpin-like folding as their lowest energy states were considered as miRNA candidates. A sequence alignment search against non-coding RNAs collected in Rfam [51] using BLASTN (version 2.2.6) was applied to identify and remove homologs of non-coding RNAs other than miRNAs. The remaining 95 sequences were retained as our final miRNA dataset.

### Northern blot hybridizations

Two-day-old seedlings, 4-week-old adult plants, root-regenerated calluses and mixed-stage flowers of *A. thaliana *(Col-0) were used to extract total RNA using the trizol reagent (Invitrogen). Samples of 20 μg total RNA were resolved in a 15% polyacrylamide/1x TBE/8 M urea gel and blotted to a zeta-probe membrane (BioRad). DNA oligonucleotides with the exact complementary sequence to candidate miRNAs were end-labeled with [γ-^32^P]ATP and T4 polynucleotide kinase (New England Biolabs) to generate high specific activity probes. Hybridization was carried out using the ULTRAHyb-Oligo solution according to the manufacturer's directions (Ambion), and signals were detected by autoradiography.

### Finding MPSS evidence for miRNA candidates

To obtain genomic regions corresponding to miRNA precursors, we extracted 500 nucleotides upstream and downstream of every genomic locus of all known and predicted *Arabidopsis *miRNAs. If an intergenic region encoding a miRNA had fewer than 500 nucleotides on either side of the miRNA locus, sequences were extracted up to the neighboring gene.

Two *Arabidopsis *MPSS datasets were used in this study: a MPSS database from abscisic acid (ABA)-treated plants, and plants with elevated levels of endogenous cytokinin [43,44] and a second public MPSS dataset produced by the Meyes laboratory at the University of Delaware, which covers gene-expression information for five *Arabidopsis *tissues at different developmental stages - around 10-week-old active growing calluses initiated from seedlings, mixed-stage buds and immature flowers, 14-day-old leaves, 14-day-old roots and 24- to 48-h post-fertilization siliques [42].

### miRNA target gene prediction and 5' RACE

miRNA target gene prediction was performed by aligning miRNA sequences with target mRNA sequences using the TimeLogic implementation of the Smith-Waterman nucleotide-alignment algorithm. Sequences of known and predicted *Arabidopsis *miRNAs and their *O. sativa *orthologs were used as query datasets. mRNA sequences of the *Arabidopsis *and *O. sativa *annotated genes were used as target datasets. Gaps were allowed in the pairing of miRNA and their target mRNAs. Mismatches were preferred over gaps by assigning higher penalties to gaps in the alignment algorithm. Consecutive gaps were preferred over scattered individual gaps by assigning higher penalties to gap opening than to gap extension. The top 500 putative hits from *Arabidopsis *miRNA target list and their *O. sativa *ortholog target list were compared. For each mRNA hit of an *Arabidopsis *miRNA, if a rice ortholog from the same gene family was also found among the top 500 rice miRNA hits, the *Arabidopsis *mRNA hit was selected as a putative miRNA target.

Tissue comparison (reference vs flower) microarray data used for target gene validation were downloaded from TAIR [46]. mRNA samples from whole plants and flowers were used as reference and sample probes in the microarray hybridization.

To identify the products of miRNA-directed cleavage we used the First Choice RLM-RACE Kit (Ambion) in 5' RACE experiments, except that we used total RNA (2 μg) for direct ligation to the RNA adaptor without further processing of the RNA sample. Subsequent steps were according to manufacturer's directions. Oligonucleotide sequences for PCR amplification of At3g26810, At1g12820 and TIR1 (At3g62980) are available upon request.

### miRNA clustering and alignment

Predicted miRNAs were compared to known *Arabidopsis *miRNAs using the MEME motif-searching software and Smith-Waterman gapped local alignment to identify homologs of known miRNAs. Pairs of aligned sequences were grouped by transitive closure, and multiple alignments were generated with ClustalW [52-54]. The multiple alignment output was manually curated.

### Free energy calculation

We used mfold program to calculate the free energy (ΔG) of predicted miRNA::mRNA pairs. For each miRNA::mRNA pair, the miRNA sequence was linked by 'LLL' to the target mRNA sequence. The 'LLL' linker sequence tells the mfold program to treat the miRNA and target sequence as two separate RNA sequences for energy calculation [38].

## Note added in proof

During revision of this manuscript, three groups [55-57] reported novel *Arabidopsis *miRNAs, some of which are included among the predicted miRNAs in this work, confirming the validity of our approach.

## Additional data files

The following additional data are available with the online version of this paper: the complete list of predicted miRNAs (Additional data file 1); a full list of the secondary structures of predicted precursors of *Arabidopsis *miRNA candidates and their rice orthologs (Additional data file 2); MPSS evidence for known and predicted *Arabidopsis *miRNAs (Additional data file 3); a complete list of predicted target mRNAs and their pairing with miRNA sequences (Additional data file 4).

## Supplementary Material

Additional data file 1 The complete list of predicted miRNAs Click here for additional data file

Additional data file 2 A full list of the secondary structures of predicted precursors of *Arabidopsis *miRNA candidates and their rice orthologs Click here for additional data file

Additional data file 3 MPSS evidence for known and predicted *Arabidopsis *miRNAs Click here for additional data file

Additional data file 4 a complete list of predicted target mRNAs and their pairing with miRNA sequences Click here for additional data file

## Figures and Tables

**Table 1:** miRNAs verified by northern blot hybridizations and their supporting evidence

miRNA name	Sequence	NB	MPSS	ASRP
miR171b	CGAUUGAGCCGUGCCAAUAUC	+	+	NA
miR413	AUAGUUUCUCUUGUUCUGCAC	+	+	NA
miR414	UUCAUCUUCAUCAUCAUCGUC	+	+	NA
miR415	AACAGAGCAGAAACAGAACAU	+	+	NA
miR416	UGAACAGUGUACGUACGAACC	+	NA	NA
miR417	UGAAGGUAGUGAAUUUGUUCG	+	NA	NA
miR393a	UCCAAAGGGAUCGCAUUGAUC	+	NA	NA
miR418	UAAUGUGAUGAUGAACUGACC	+	+	NA
miR419	UUAUGAAUGCUGAGGAUGUUG	+	+	NA
miR169b	CAGCCAAGGAUGACUUGCCGG	+	NA	+
miR396b	UUUCCACAGCUUUCUUGAACU	+	NA	NA
miR420	UAAACUAAUCACGGAAAUGCA	+	+	NA
miR169g*	UCCGGCAAGUUGACCUUGGCU	+	NA	NA
miR172b*	GCAGCACCAUUAAGAUUCAC	+	+	+

NB, northern blot hybridization; MPSS, massively parallel signature sequence; ASRP, sequence present in the *Arabidopsis *Small RNA Project database; NA, data not available.

**Table 2:** Analysis of predicted miRNA target functions using GO annotation

Molecular function	Number of putative targets
Antioxidant activity	17
Nucleic acid binding	80
Catalytic activity	152
Enzyme regulator activity	4
Signal transducer activity	51
Structural molecule activity	1
Transcription regulator activity	129
Translation regulator activity	27
Transporter activity	41
Targets with GO annotation	254
Total predicted targets	371

**Table 3:** Family specificity of putative miRNA-targeted transcription factors

Transcription factor gene family	Predicted number of proteins*				Predicted number of miRNA targets	Percent members targeted^†^
			
	*Arabidopsis thaliana*	*Drosophila melanogaster*	*Caenorhabditis elegans*	*Saccharomyces cerevisiae*		
MYB superfamily	190	6	3	10	22	11.6%
bHLH	139	46	25	8	3	2.2%
HB	89	103	84	9	4	4.5%
MADS	82	2	2	4	4	4.9%
bZIP	81	21	25	21	4	4.9%
CCAAT	36	7	7	6	1	2.8%
AP2	14	0	0	0	6	42.9%
NAC	109	0	0	0	10	9.1%
WRKY	72	0	0	0	1	1.4%
GRAS	32	0	0	0	9	28.1%
SBP	16	0	0	0	8	50.0%

*Data in this column are taken from [58]. ^†^The percentage of transcription factors in each family targeted by miRNA in *Arabidopsis*.

**Table 4:** Flower microarray expression data for putative targets of miRNAs identified by northern blot hybridization

miRNA	Target description	Target ID	Expression change
miR414	DEAD box RNA helicase	At1g20920	-1.10 fold
	F-box protein family	At1g15670	-1.06 fold
		At2g44130	-1.98 fold
	SNF2domain/helicase domain-containing protein	At3g42670	-1.29 fold
	Nucleosome assembly protein	At4g26110	-1.37 fold
miR393a	F-box protein family	At3g62980	-1.07 fold
		At3g26810	-1.67 fold
miR419	Histidine kinase	At2g01830	-3.35 fold
miR169b	CCAAT box binding factor	At5g12840	-2.60 fold
miR396b	Transcription activator	At2g36400	-1.51 fold
		At4g37740	-2.08 fold

**Table 5:** Summary of evidence for predicted miRNAs

Category	Number of miRNA candidates
Total predicted miRNAs	95
Previously known miRNAs included	12
Newly predicted miRNAs	83
Northern blot positive	12 (14 total)
cDNA (ASRP)	8
MPSS	16
Known *Arabidopsis *miRNA homologs	8
Northern blot and MPSS	8
ASRP and MPSS	3
Unique miRNAs with northern blot hybridization, ASRP or MPSS evidence	25
miRNAs with functionally conserved targets in *Arabidopsis *and rice	77
