# Supplementary material for: Prediction and identification of Arabidopsis thaliana microRNAs and their mRNA targets
Source: Genome Biol. 2004 Aug 31;5(9):R65. doi: 10.1186/gb-2004-5-9-r65 (PMC522872; doi:10.1186/gb-2004-5-9-r65)
Supplement: Additional data file 1 — The complete list of predicted miRNAs [file gb-2004-5-9-r65-s1.doc]

**Additional datafile 1.** List of predicted *Arabidopsis* miRNAs and their supporting evidence. NB, Northern blot hybridization positive; MPSS, evidence from massively parallel signature sequencing; ASRP, cDNA evidence from the *Arabidopsis* Small RNA Project (ASRP) database; HAM, homolog of known *Arabidopsis* miRNAs.

miR name Sequence miR position NB MPSS ASRP HAM

miR171b CGAUUGAGCCGUGCCAAUAUC Chr1, ds of At1g11730(a) + + NA +

miR413 AUAGUUUCUCUUGUUCUGCAC Chr1, ds of At1g62350(s) + + NA -

miR414 UCAUCUUCAUCAUCAUCGUCA Chr1, ds of At1g67190(a) + + NA -

miR415 AACAGAGCAGAAACAGAACAU Chr1, ds of At1g74450(s) + + NA -

miR416 GGUUCGUACGUACACUGUUCA Chr2, ds of At2g16140(a) + NA NA -

miR417 GAAGGUAGUGAAUUUGUUCGA Chr2, ds of At2g32270(a) + NA NA -

miR393a UCCAAAGGGAUCGCAUUGAUC Chr2, ds of At2g39880(a) + NA NA -

miR418 UAAUGUGAUGAUGAACUGACC Chr3, ds of At3g18890(a) + + NA -

miR419 UUAUGAAUGCUGAGGAUGUUG Chr4, ds of At4g32440(s) + + NA -

miR169b CAGCCAAGGAUGACUUGCCGG Chr5, ds of At5g24820(s) + NA + +

miR396b UUUCCACAGCUUUCUUGAACU Chr2, ds of At5g35400(a) + NA NA -

miR420 UAAACUAAUCACGGAAAUGCA Chr5, ds of At5g62840(s) + + NA -

miR169g* UCCGGCAAGUUGACCUUGGCU Chr4, ds of At4g21590(a) + NA NA -

miR169h UAGCCAAGGAUGACUUGCCUG Chr1, ds of At1g19370(a) - NA + +

miR395a UGAAGUGUUUGGGGGAACUCC Chr1, ds of At1g26970(a) - NA NA -

At1g36990_5_30 UUUUGGUUUGGACAGUUGUUU Chr1, ds of At1g36990(a) - NA NA -

At4g31350_5_17 UUGAGACUUGAGACUGAACAU Chr4, ds of At4g31350(s) - + NA -

At1g03620_1175_1 UAGUGAUCUGAUUGGUUGAGG Chr1, ds of At1g03620(a) NA NA NA -

At1g11380_95 UUGGUCUUGUUCAGUUCUGUU Chr1, ds of At1g11380(a) NA + NA -

At1g13250_7364_1 CUCUAAUUGGCUCUUUGCAUA Chr1, ds of At1g13250(s) NA NA NA -

miR399a UCUGCCAAAGGAGAUUUGCCC Chr1, ds of At1g29260(s) NA NA NA -

At1g32200_463_rc GUUGUCGACGUCACAUGAUUU Chr1, ds of At1g32200(a) NA NA NA -

At1g38122_654_rc UCAGAUGAAGAUGGAGAUGGA Chr1, ds of At1g38122(a) NA NA NA -

At1g41790_1104_1 GCCAUCUCCUCGUCAUUGUGC Chr1, ds of At1g41790(s) NA NA NA -

At1g42080_527 GAGUCUCAAUAGGAAUUUAGA Chr1, ds of At1g42080(a) NA NA NA -

At1g53360_1326_rc GCAGAGACUAUGUUUGGUUUG Chr1, ds of At1g53360(s) NA NA NA -

miR169d UGAGCCAAGGAUGACUUGCCG Chr1, ds of At1g53680(a) NA NA + +

At1g61840_4217 CCAUGACUCUCUUUUUCUUCU Chr1, ds of At1g61840(a) NA NA NA -

At1g66810_329_1 GGUUUUGUUUGUAACUUGGAG Chr1, ds of At1g66810(a) NA NA NA -

At1g73320_140_rc UGCUUGAGAGUUGAGACAA Chr1, ds of At1g73320(a) NA NA NA -

At1g75680_601 GGUGGUUCAUUGUUGUUGCCA Chr1, ds of At1g75680(a) NA NA NA -

At3g08760_3637_rc AUGCAGAUUUUGCAUUUG Chr3, ds of At3g08760(a) NA NA NA -

At4g11130_6601_rc AGUGAUGGCCAUGGCAUGGAA Chr4, ds of At4g01450(a) NA NA NA -

At4g12190_99_rc UCUCUCUGUUUUGCAUUAUCA Chr4, ds of At4g01450(a) NA NA NA -

At4g13550_1386 UCAACGAUGCACUCAAUGAUG Chr4, ds of At4g13550(a) NA NA NA -

At4g16100_467 UGUAUGCGUGUUAUGGAUAUG Chr4, ds of At4g16100(s) NA NA NA -

At4g29200_13 UCACAACUCUCAAGGUUUUGU Chr4, ds of At4g16100(s) NA NA NA -

At4g38520_371_1 GGACGAGAGGGAAGUGGAGCC Chr4, ds of At4g38520(s) NA NA NA -

miR172b* GCAGCACCAUUAAGAUUCAC Chr5, ds of At5g04270(s) + + + -

**miR398b**  UGUGUUCUCAGGUCACCCCUG Chr5, ds of At5g14540(s) NA + NA -

At5g20490_133 GAGAGACCGAUUUUGCAGAAA Chr5, ds of At5g20490(s) NA + NA -

At5g25610_3237 CACUCCAAAUCUCCAACUCGU Chr1, ds of At5g25610(s) NA NA NA -

At5g38490_130_1 AAUGCAGAAAACAGUGGAGUC Chr5, ds of At5g38490(s) NA NA NA -

At5g38550_198_rc CAAAUGAUCCCUUGUCUC Chr5, ds of At5g38550(a) NA NA NA -

At5g40770_3770_rc GAAUUGUGAAUUUGUGAUCGU Chr5, ds of At5g40770(s) NA NA NA -

At5g55830_310_rc UUGACAGAAGAAAGAGAGCAC Chr5, ds of At5g55830(a) NA NA NA +

At5g56680_271 UUCUCUAUCGAUCUCCAUUGU Chr5, ds of At5g56680(s) NA NA NA -

At5g57630_760 UGUUGCUUCCUGUUUAUG Chr5, ds of At5g57630(a) NA NA NA -

miR172e GAAUCUUGAUGAUGCUGCAUC Chr5, ds of At5g59500(s) NA NA NA +

At5g62160_613_rc GGGCAACUCUCCUUUGGCAAG Chr5, ds of At5g62160(s) NA NA NA -

**miR398a**  UGUGUUCUCAGGUCACCCCUU Chr2, ds of At2g03440(a) NA NA NA -

miR172d AGAAUCUUGAUGAUGCUGCAG Chr3, ds of At3g55510(a) NA NA + +

At1g16360_5_rc CCAUUUUGUGUUGUUGGACUA Chr1, ds of At1g16360(a) NA NA NA -

At1g22640_5_13_rc UUUGUGACUUCUGAGUGAGGC Chr1, ds of At1g22640(a) NA NA NA -

At1g30825_5_4_rc CAAAAGGAGGAGUACUAUUUA Chr1, ds of At1g30825(s) NA NA NA -

At1g40136_5_24 UAUGGUUUAGGGUUUACGGUU Chr1, ds of At1g40136(a) NA NA NA -

At1g51175_5_8 UUUUUGCAGAUGAAUGAGCUG Chr1, ds of At1g51175(a) NA NA NA -

At1g60020_5_14 UUUUGGAAAUUUGUCCUUACG Chr1, ds of At1g60020(a) NA NA NA -

miR171c UUGAGCCGUGCCAAUAUCACG Chr1, ds of At1g62030(s) NA + + +

At2g05580_5_4 GUAGUCAGAGAAUUUCGAUUG Chr2, ds of At2g05580(s) NA NA NA -

At2g24430_5_9 CCGAUGUGUGAAUGUGAACAU Chr2, ds of At2g24430(a) NA NA NA -

At2g36540_5_rc UCAGAGACAAAGACCAUUAGU Chr2, ds of At2g36540(s) NA NA NA -

miR390a AAGCUCAGGAGGGAUAGCGCC Chr2, ds of At2g38320(a) NA + + -

At3g04840_5 UCUGAUUUGUGGAGUUUGUUU Chr3, ds of At3g04840(s) NA NA NA -

At3g10000_5_5 GAUUUAGCCCUUCAUGUUUAU Chr3, ds of At3g10000(s) NA NA NA -

At3g43570_5_29 GAUCUCUAUCACUUUCUCUCA Chr3, ds of At3g43570(s) NA NA NA -

At3g52510_5_6 UUUUACGGAGGUUCAAAAGCU Chr3, ds of At3g52510(s) NA NA NA -

At3g61890_5_33 UUGAGGGGACUGUUGUCUGGC Chr3, ds of At3g61890(a) NA + NA -

At3g63200_5_9 UAUGGAGAUAAGUACUAGUUG Chr3, ds of At3g63200(s) NA NA NA -

**miR397a**  UCAUUGAGUGCAGCGUUGAUGU Chr4, ds of At4g05100(s) NA NA + -

At4g10845_5_11 GCAUUCAUGCAUACAUCCAUA Chr4, ds of At4g10845(s) NA + NA -

At4g15250_5_1 UUAAUGUGUGACUGUGACUGU Chr4, ds of At4g15250(a) NA NA NA -

At4g23710_5_3_rc UGGAUGAAUGAGUCGGAAGCU Chr4, ds of At4g23710(a) NA NA NA -

At5g25180_5_12 UGUCCAUCCAUACCCAAAAAU Chr5, ds of At5g25180(a) NA NA NA -

miR164c UGGAGAAGCAGGGCACGUGCG Chr5, ds of At5g27800(s) NA NA NA +

At5g36280_5_3 GUUUGCUUGUCGCUGGCGACU Chr5, ds of At5g36280(a) NA NA NA -

At5g39200_5_10_rc UGAGUUGAGUUGAGUCGACUC Chr5, ds of At5g39200(s) NA NA NA -

At5g63700_5_1_rc UGACACAGGUCACUCAAACAG Chr5, ds of At5g63700(a) NA NA NA -

At1g25460_26 UUCACAAGUCACAAUCACCA Chr5, ds of At1g25460(a) NA NA NA -

At3g27883_1009 AGAUUCGAACUCUAGUCCUC Chr5, ds of At3g27883(a) NA NA NA -

At5g02330_2413_rc CCGAACUCAUUUUGGUUUUUG Chr5, ds of At5g02330(a) NA NA NA -

At5g32470_7631_rc GAGAAUAUGUGAGCUGGUAAA Chr5, ds of At5g32470(a) NA NA NA -

At2g28470_1616_rc UUUGUACUUUGUAGGUUUGG Chr5, ds of At2g28470(a) NA NA NA -
