# Supplementary material for: Prediction and identification of Arabidopsis thaliana microRNAs and their mRNA targets
Source: Genome Biol. 2004 Aug 31;5(9):R65. doi: 10.1186/gb-2004-5-9-r65 (PMC522872; doi:10.1186/gb-2004-5-9-r65)
Supplement: Additional data file 2 — A full list of the secondary structures of predicted precursors of Arabidopsis miRNA candidates and their rice orthologs [file gb-2004-5-9-r65-s2.doc]

**Additional File 2. Structures of predicted *Arabidopsis* microRNA precursors**

**>miR171b CGAUUGAGCCGUGCCAAUAUC**

***Arabidopsis thaliana miRNA precursor:***

UAUGCAAGGUAA A A UG A -- C UAA

UGGUUU CGCG GAUAUU G CGGUUCAAUC AAUAG UCGU CUCU C

ACCAAA GCGC CUAUAA C GCCGAGUUAG UUGUU GGCA GAGG U

UG---------- A C GU C GU A UAC

***Oryza sativa miRNA precursor:***

U G U U C C UGGC U A--- C

AUGAAA GGUA CUA GAUGUUGGC CGGCUCA UCAGA GGCAU GUGA GC AAGCAUG A

UACUUU UCGU GAU CUAUAACCG GCCGAGU AGUCU UUGUG CACU CG UUCGUGC U

C - U U U - UU-- - AUCG G

**>At1g16360_5_rc CCAUUUUGUGUUGUUGGACUA**

***Arabidopsis thaliana miRNA precursor:***

GU- C ACUU GUUUUCU .-U -- GU

AUAUUAGUCUA ACACA AAAUGGGU GC UCUUUGUU GG UAU U

UGUAAUCAGGU UGUGU UUUACCCA UG GGGAACAA CC AUG C

UGU - AAGU AUGU--- \ - UA AU

A--- A

UUUC U

AAAG G

UUUG U

***Oryza sativa miRNA precursor:***

- CU GC UU UCG

UAG CCA AGCA CAG UGAGG \

AUC GGU UUGU GUU ACUUU U

A UU -- UU UUG

**>At1g22640_5_13_rc UUUGUGACUUCUGAGUGAGGC**

***Arabidopsis thaliana miRNA precursor:***

AC UGU-- - AA - - GAAA AAAA

UUAC CUU UUU CCUC ACUCGGAAG CA AAGA UGGAU G

AAUG GAG GAA GGAG UGAGUCUUC GU UUUU AUCUA C

A- UAGAU C -- A G AUAA AAAU

***Oryza sativa miRNA precursor:***

- CA- - - - AAU U CUU UU

AUG AACCG GCAUUUA UCA AAG CACAAAC--GC AGACG AUU GC U

UGC UUGGU UGUGAGU AGU UUC GUGUUUG CG UCUGC UGA CG G

A UUG G C A \ AAU - AAU UA

A--- UUAUUU

GUUUUA U

CGAGAU A

UACA UCUUUA

**>miR395a UGAAGUGUUUGGGGGAACUCC**

***Arabidopsis thaliana miRNA precursor:***

G UC UA UG U GAUACA U

UU GUUGGAUG UCC GAGUUCCUC AGCACUUCA UGGG AUUU U

AG CAACUUAC AGG CUCAAGGGG UUGUGAAGU ACCU UAAA C

G CC CC GU C AUUAG- U

***Oryza sativa miRNA precursor:***

U C - --- U C UACAAU

GUUGG UGUUACCUGGAGUUUCCUCAA ACACUUCAC AUC UGC AGGC CUAU U

CGAUC ACGGUGGAUCUCAAGGGGGUU UGUGAAGUG UGG ACG UCUG GGUG G

C - A UUA - A UAACGC

**>At1g30825_5_4_rc CAAAAGGAGGAGUACUAUUUA**

***Arabidopsis thaliana miRNA precursor:***

A U ACG AUA AUUACCACAAACUC UUAA

U AAAUAGU CUC CUUU UGG ACAU \

A UUUAUCA GAG GGAA ACC UGUA A

- U GA- A-- GAACCAUUUUUUU- UUUU

***Oryza sativa miRNA precursor:***

-- C -- ----- - G ACCAAC U

CCAA CAGAA AGU UUCUCUUUUUU UUUUC UG AUUUG UGAAUA \

GGUU GUUUU UCA AGGAGGAAAAG AAAAG AU UGAAC ACUUAU C

UC A UG AUAUA C G AGAGAC U

**>At1g36990_5_30 UUUUGGUUUGGACAGUUGUUU**

***Arabidopsis thaliana miRNA precursor:***

.-UA UU G AC U CA- .-A UA

CAUUAG UAUU UUUUG UUUGG AG UGUUU UUUUAG ACA \

GUAAUC AUAA AAAAC AAACC UC ACAAA AAAAUC UGU A

\ -- UC - A- C AAA \ - UG

G-- U

GA G

CU U

AUU A

***Oryza sativa miRNA precursor:***

ACA --- G GG .-G U- CC

GCAU AGGUCCCA AUUU GUUUGGAC UUGUUUU GUU CGUC A

UGUA UCCGGGGU UAAA CAAACUUG AAUAAAA CAG GCAG C

CA- GUA A AA \ - UU UA

AUG---------- AU

GC A

CG G

AGAAAAUUUAGAA AA

**>At1g40136_5_24 UAUGGUUUAGGGUUUACGGUU**

***Arabidopsis thaliana miRNA precursor:***

AU GGG A U GG U A .-GGUUUA GG GG UAA

AGUUU GGUUUA UUU CGGU UU GUU AGG UUUAG G GUUA G A

UCAAG CCAGGU GAA GCCA AG CAA UCC AGAUC C UAGU U G

-- GA- - U AA C A \ ------ UU AU UGA

A-- AGG

UGUGU A

AUAUA U

AAA AGU

***Oryza sativa miRNA precursor:***

GG- UA UG

GUAUGGUUUAGGGUUUA GUU GCC G

CGUACUAAAUUCCAGGU CGA CGG U

AAG -- UU

**>At1g51175_5_8 UUUUUGCAGAUGAAUGAGCUG**

***Arabidopsis thaliana miRNA precursor:***

UU C U U C G

UUUUU UUG AGA GAA GAG UGAAU \

AAAAA AAC UUU UUU UUC AUUUG A

UC U U U A U

***Oryza sativa miRNA precursor:***

U UC U U GAUGAA AAGA

AUUGU UGC UUUU UUUU UGCU UGAGCUG \

UAACA AUG AAAA AAAG ACGG ACUUGAC U

C U- C C ------ AAGG

70 60 50

**>At1g60020_5_14 UUUUGGAAAUUUGUCCUUACG**

***Arabidopsis thaliana miRNA precursor:***

GG- GACA GGAA- U A .-GU CU

GAG GG AUUUUU AU UGUCCUU CGG AGUA A

CUC UU UAAAAA UA ACAGGAA GCC UCAU G

AAA AAAA AGGAG U - \ -- AA

U----------- CA

GUC \

CAG C

AAUAAAGUUUAG UA

***Oryza sativa miRNA precursor:***

UUUG UUU CC AG .-CAAAA AG AAU A AA

UCGCAUU GAAG GU UUACGAAA GCUAG UUUAGUG GC GA UG A

AGCGUAA CUUC UA AGUGCUUU CGGUU AAAUCAU CG CU AC A

---- C-- AA A- \ ----- AG AU- - AG

AUUAAAA ACCCU

UUUGAUA \

AAACUGU A

UC----- GUAAA

**>miR171c UUGAGCCGUGCCAAUAUCACG**

***Arabidopsis thaliana miRNA precursor:***

UA A UG - UAC UG

AAA CG GAUAUUGG CGGUUCAAUCAGA AAACCG UCUUU U

UUU GC CUAUAACC GCCGAGUUAGUUU UUUGGC AGAAA U

GC A GU A U-- UU

***Oryza sativa miRNA precursor:***

U U C C UGGC U A--- C

GCUA GAUGUUGGC CGGCUCA UCAGA GGCAU GUGA GC AAGCAUG A

UGAU CUAUAACCG GCCGAGU AGUCU UUGUG CACU CG UUCGUGC U

U U U - UU-- - AUCG G

**>miR413 AUAGUUUCUCUUGUUCUGCAC**

***Arabidopsis thaliana miRNA precursor:***

CC UCUUG CAU UAA AGGAACCAUG G -- AG

GAU AUAGUUUC UUCUGCA CCAC CUUC UCCCA UU UC G

CUG UAUCAAGG AAGACGU GGUG GAAG AGGGU AA AG U

CU UCA-- AUC UUA GUAAACAAA- G CU AU

***Oryza sativa miRNA precursor:***

---A A AA AGUUAGC UA

UG GGA AC ACUC \

AC CCU UG UGAG U

ACGA - AA C------ UC

AC ---A C G A AC UC / -- ACA

GC UCC A G CU GUUUC UUGU UGCAC UUAUU \

CG AGG U C GA UGAAG AACA ACGUG AAUAA G

-A AGUC A A C -A UU .-AA AAA

**>miR415 AACAGAGCAGAAACAGAACAU**

***Arabidopsis thaliana miRNA precursor:***

AC A -- .-AUAUUCUC UUUU

AGA AGAGC GAAAC AGAACAU UGUC \

UCU UCUCG UUUUG UCUUGUA ACGG U

CU A GU \ -------- UGUU

AAAGUAA CG C

AUGG AGAAGA A

UACC UUUUCU A

AGC---- A- C

***Oryza sativa miRNA precursor:***

AGAA A -- C -- U

GC ACAGA CAG AAG AGAGC AGCU--GUG A

CG UGUCU GUC UUC UCUCG UCGA CAC U

AACA - GG C AG \ A

AACA- AACUA AUC

CUGC UCCCU \

GACG AGGGA A

CCAAA AAAC- GUC

**>At2g05580_5_4 GUAGUCAGAGAAUUUCGAUUG**

***Arabidopsis thaliana miRNA precursor:***

CCA CA CGA GA -------------- UGC

UUUCAGA GUAGU GAGAAUUU UUG GCAAUGA AUGAGGAA U

AAAGUUU UAUCA UUCUUAAA AAC CGUUAUU UAUUUUUU A

--- -- AAC AA UCAAUGUAAAGUUU UUC

***Oryza sativa miRNA precursor:***

U U - G CUC-- -------------- ACA U U UCU UU

GAAG AAG CA GA AAU GAUUGGUACA UCUCC CG AA AGGA UUA \

CUAC UUU GU CU UUA CUGAUCAUGU AGAGG GU UU UUUU GAU A

- - A G UCCGU UUAUGAAGGUUGGG GGG U U UU- UA

**>At2g24430_5_9 CCGAUGUGUGAAUGUGAACAU**

***Arabidopsis thaliana miRNA precursor:***

---- G AAU A - U U G AU

GAUGA CC AUGUGUG GUGAACAUU AC CGA UUG GU AAU G

CUACU GG UACGUAU CACUUGUAA UG GUU AGC CA UUA A

UACA - AU- G U U - A CA

***Oryza sativa miRNA precursor:***

C GG G AAU A - AAUUCA AGAAGCAGCA AC

ACAUGCC UG CC AUGUGUG GUG A C CCUAGCU UCC \

UGUAUGG AU GG UACACGU CAC U G GGAUCGA AGG U

- A- - ACU G A CGACAA AAAGAAAA-- AC

**>miR417 GAAGGUAGUGAAUUUGUUCGA**

***Arabidopsis thaliana miRNA precursor:***

U AA- GG- AA CG- UUU UC CUU GA

AAAUAUAU CAA GU UC AACA UCACUA CCUUUAUGUUU CC AUU U

UUUAUAUA GUU UA AG UUGU AGUGAU GGAAGUACAAA GG UAA G

- GUA AUA C- UUA --- UU --- AG

***Oryza sativa miRNA precursor:***

U U GA--- UUAA UCUUA

U UGG ACAAAUUU AUUC AUC \

G ACC UGUUUAAG UAAG UAG U

U U UGAUG UA-- UAUUA

**>At2g36540_5_rc UCAGAGACAAAGACCAUUAGU**

***Arabidopsis thaliana miRNA precursor:***

UU ACUU CU U CUAA- A UU- - UUU

UCUUUUU GU AU UUAAU GUUUUU UGACU UCUUU UCA C \

GGGAAAA CA UA GAUUA CAGAAA ACUGA AGAAG AGU G U

UU GU-- AU C CAGAG G CUU U UCU

***Oryza sativa miRNA precursor:***

- UUA- A- A CGAG AUU

CU UUAGUAAUUCAUUUACUA GU UGUUUUUGAUA GUAAU UUACU \

GA AAUCAUUGAGUAAAUGAU CG ACAGAGACUAU CAUUG AAUGA C

C UACC AA - AGUA GCU

**>miR390a AAGCUCAGGAGGGAUAGCGCC**

***Arabidopsis thaliana miRNA precursor:***

-- AU U A G --------- UC

GUAG AGAAGA C GU AAGCUCAGGA GGAUAGCGCCA UGAUGA A

CAUC UCUUCU G UA UUUGAGUCCU CCUAUCGCGGU AUUGCU C

AU CG U C A UUUUUAUCU UA

***Oryza sativa miRNA precursor:***

U U U A G CGAAA

GGUA GGAACAA CC UG AGCUCAGGA GGAUAGCGCCU U

CCAU UCUUGUU GG AC UCGAGUCCU UCUAUCGCGGA C

- U U C A UCAAA

**>At3g04840_5 UCUGAUUUGUGGAGUUUGUUU**

***Arabidopsis thaliana miRNA precursor:***

UAAUAUCAAGAA GAU GG G

UUCCG UCU UUGU AGUUUGUUU A

GAGGC AGA AACG UUGAGUAAG A

UAAAACAAGAG- ACC A- A

***Oryza sativa miRNA precursor:***

GA UG -- - CUA

GGCU UUUG GAGUU UUUUUGG GU \

UCGG AGAC UUCAA AGAAACC CA A

AA UU UG A UAU

**>At3g10000_5_5 GAUUUAGCCCUUCAUGUUUAU**

***Arabidopsis thaliana miRNA precursor:***

- A CU- U A - U A A A

GAG CU UGGG UUG GAUUU GCC CUUCA GUUU UAAAU--GGG UGUG A

CUC GA ACCC GGC CUAAA CGG GGAGU CGAA AUUUA CCC ACAC U

U - CUU U A C - A \ G U

ATCA-------------- ACAAT

TGAA \

ACTT A

GGAACAAAACAAAAACGA AATAT

***Oryza sativa miRNA precursor:***

GC - AGUU UU - UUCC A U .-CACG GG AC

GU UC AUGAGU GA UAGC CC UGUUU AUU ACAUC AU GCU \

CA AG UACUCG CU GUUG GG ACAAA UGA UGUAG UG UGG A

AA A GU-- UC U UA-- C U \ ---- UU AU

------ UU

GAG \

CUC C

CUCAAA AA

**>At3g43570_5_29 GAUCUCUAUCACUUUCUCUCA**

***Arabidopsis thaliana miRNA precursor:***

--- UCUU- UCACU U ACG CUAACCACUA UA

AAUCUUC UGUU GAUCUCUA UUC CUC CAU AUUUCAUC U

UUAGAAG ACAA CUAGAGGU AAG GAG GUA UAAAGUAG C

UUA CUUUU UUC-- - GA- ---------- UC

Oryza sativa miRNA precursor:

A- U A - - GAUG AUGA - AGCA- AC--- U UG

UCAG UC CCAUC CUUU CUCUC UC CCUCG UC GCU GAGCC CUCCA C \

AGUC AG GGUGG GAGG GAGAG AG GGAGC AG CGA CUCGG GAGGU G C

CG - A C G AUGA ---- U CAGAA AAGAA C AG

**>At3g52510_5_6 UUUUACGGAGGUUCAAAAGCU**

***Arabidopsis thaliana miRNA precursor:***

A C U AAA UUAAUUCUACUC

AG UA AUUUUACGGAGGU CA GC U

UC GU UAAAGUGUCUCCA GU CG U

- U - A-- CAAAAUAUCUAA

***Oryza sativa miRNA precursor:***

.-AAAUAACA - AC - AAA AG

GGGA CCGC UUUU GGAGG UUUA GCUAA C

UCCU GGUG AAAA UCUCC GAAU UGGUU U

\ -------- U -- U C-- AC

70 60 50

**>At3g61890_5_33 UUGAGGGGACUGUUGUCUGGC**

***Arabidopsis thaliana miRNA precursor:***

UUUUUCUU A C UU CU G U U-- A - -- U

GAGGGA UUG GGGGA UG GUCUGG CGA GAC CU AUUCUAAU CAAUC UCA UUUGAA A

CUCCCU AAC CCCCU AC CGGACC GCU CUG GA UGGGAUUA GUUAG AGU AGACUU C

CACUAUU- C U UU AG G U UUU - U CU A

***Oryza sativa miRNA precursor:***

A UUC U UU CU G .-U A

GGA GCUUU ACUU GAGGGGAAUG GUCUGG CGAGGU CA GGAG A

CCU CGAAA UGAA CUCCCCUUAC CGGACC GCUCUA GU UCUC A

- --- - UU AG - \ - C

--------- UC

GAUCGA A

CUAGUU G

CAGAGAUGU UG

**>At3g63200_5_9 UAUGGAGAUAAGUACUAGUUG**

***Arabidopsis thaliana miRNA precursor:***

UG- A UACU UGG GAC - U C

AGAUUAAUUA GAG UAAG AGUUGG GA AC UG UCAUUAU \

UCUAAUUAAU UUC AUUC UCAACU UU UG AC AGUAGUA U

UAA - UUU- UA- AA- C C G

***Oryza sativa miRNA precursor:***

GUAUGUUU AGA AG CU --- GAUAU GGCUUUU U

GCGU UUGUGG UA UA AUUUGGU UGAUGUUG GCCUU GGAU U

CGCA AACACC GU AU UAGGCCG ACUAUAGC UGGGA UCUG A

AAUCCU-- A-- GA U- UGU AAC-- AUAUCCC U

**>miR397a UCAUUGAGUGCAGCGUUGAUGU**

***Arabidopsis thaliana miRNA precursor:***

UUU-- U C A U G UUU G

UUCCUGGG GAA GAACAU AUUGAGUGCAGCGUUG UGUAA UUC UUUUG UUCAUU U

AAGGACUU CUU UUUGUA UAACUCGCGUUGCGAC AUAUU AAG AAAAU AGGUAA U

UUAAU - U C U - U-- G

***Oryza sativa miRNA precursor:***

A- C C ACAA UA- C- UG

CAGAGA UCAAAUGCAU AUUGAGUG AGCGUUGAUGA CGG ACCGGUC AUGU A

GUCUCU GGUUUGCGUA UAACUCAC UCGCGACUACU GUC UGGCCGG UACG U

AC C U ACUA UAG UU CG

**>At4g10845_5_11 GCAUUCAUGCAUACAUCCAUA**

***Arabidopsis thaliana miRNA precursor:***

UC C AC C AC A CC C ACAU

UGCAU AUG AU AU CAU AUGCAU UAU AUA AUGCAU C

ACGUA UAC UA UA GUA UACGUA AUA UAU UACGUA C

CA C CA C CU C CC A CAUA

***Oryza sativa miRNA precursor:***

GG A A U ACA AAG AUAAU CAU UAUC

AUU AGUAA UAGAC UUGAUGCAUACA CCAU CAC UAUUU CCA GCAA \

UAA UCGUU GUUUG AGCUACGUAUGU GGUA GUG AUAAA GGU CGUU U

GA - - - GGG --- GAAUC UAU UAGU

**>At4g15250_5_1 UUAAUGUGUGACUGUGACUGU**

***Arabidopsis thaliana miRNA precursor:***

AUC AUG C GA G U---- U

GGUUCAUGU UUUUUA UGUGA UGU CU UGAC AAUACA G

UUAGGUGUA AAGAAU ACACU ACA GA AUUG UUGUGU G

A-- CAA C AA G UAAGU U

***Oryza sativa miRNA precursor:***

UGU UGU GA UGUUAAUAUAAUCU

CUCCUUAA GGUAG GACU UUCUAA \

GAGGAAUU UUGUU CUGA AAGAUU U

CU- UCU -- UGCUCUACUAAUCU

**>miR169g* UCCGGCAAGUUGACCUUGGCU**

***Arabidopsis thaliana miRNA precursor:***

AU U UU A UUUUUU AU UAAU

GGAAGA AGAGAA GAGG GAGCCAAGG UGACUUGCCGGG UACCA GAAUC \

UUUUCU UCUCUU CUUU CUCGGUUCC GUUGAACGGCCU GUGGU CUUAG U

-- C GU A ------ -- UCAA

***Oryza sativa miRNA precursor:***

U GA C UG UAC AU UU .-AA UA

GAGA AGAGAACG AUG AGCCAAGGA ACUUGCCGG GUGU GCAUG UC GGUAC \

CUCU UUUCUUGU UGC UCGGUUCCU UGAACGGCC UACA CGUAC AG CCGUG U

U UC A GU UUU GU CU \ -- UA

CCC CU

AA \

UU G

A-- UU

**>At4g23710_5_3_rc UGGAUGAAUGAGUCGGAAGCU**

***Arabidopsis thaliana miRNA precursor:***

C U U A CA UU A UA UGA

AUGAU CAAUU G CU CCG UCAUUCAUUCA UA CGAGUC GUU A

UACUA GUUAA C GA GGC AGUAAGUAGGU AU GCUCAG CGG U

A U - A UG UU G -- UUU

***Oryza sativa miRNA precursor:***

C G A- AAC C C C C UU---- AU

CGG GC AG UCGG GUUA CU CCG UUCAUUCACUCAU UGCUAAGC CGCU C

GCC CG UC AGCU UAAU GA GGC GAGUAAGUGGGUG ACGGUUCG GCGA U

A A CC --- C C U U UUCUUU AC

**>At4g31350_5_17 UUGAGACUUGAGACUGAACAU**

***Arabidopsis thaliana miRNA precursor:***

UU U UU U U---- U CCC GG

GUCU AGA UGAGAC GAGACUGAACAU GAUCC UCU GGUA UC \

CAGA UUU ACUUUG CUUUGGCUUGUA CUAGG AGA CUAU AG C

UU C UU - UAUAC U AAA UU

***Oryza sativa miRNA precursor:***

A--- A .-CAAAGCUUU C

CUUG GACUUG GAGUGAA GUCUUA U

GAAC UUGAAC CUUACUU UAGAGU A

ACAG - \ --------- G

UA-- UACG A AU

UAGUUAGU CG UU C

AUUAGUCA GC AG U

CAUA CA-- - GU

**>miR419 UUAUGAAUGCUGAGGAUGUUG**

***Arabidopsis thaliana miRNA precursor:***

A A G G AUUA--- G GUC U

AAAUU UGA UGCUGAG AUGUU UU CGAGCAAU AGAU UUUUU U

UUUAA ACU ACGAUUC UACAG AG GUUCGUUG UUUA AAAAA A

- - - G AACGUUC G AAA A

***Oryza sativa miRNA precursor:***

UCUCUGU U AU- UGACGAU GUG UUUUUU

UCU UCUCUG GAUGA GC GUUGUAGAU AAC \

AGA AGAGGU CUGUU CG UAGUAUCUG UUG U

UGUUAU- C GUU UAAG--- --- UCUCUU

**>miR169b CAGCCAAGGAUGACUUGCCGG**

***Arabidopsis thaliana miRNA precursor:***

U U C - UG CGU AAC G A GU

GGAG AGAGUA AAUG AGCC AAGGA ACUUGCCGGAA UGUU CAU CAUAUGA UAAU G

CUUC UCUCGU UUAC UCGG UUCCU UGAACGGUCUU ACAA GUA GUGUAUU AUUA A

- U A C GU UAU --- - A GU

***Oryza sativa miRNA precursor:***

U GA C UG UAC AU UU .-AA UA

GAGA AGAGAACG AUG AGCCAAGGA ACUUGCCGG GUGU GCAUG UC GGUAC \

CUCU UUUCUUGU UGC UCGGUUCCU UGAACGGCC UACA CGUAC AG CCGUG U

U UC A GU UUU GU CU \ -- UA

CCC CU

AA \

UU G

A-- UU

**>At5g25180_5_12 UGUCCAUCCAUACCCAAAAAU**

***Arabidopsis thaliana miRNA precursor:***

UC G UCC GA----- UGUUUUU- UUCG U UU

AUU UCAU UCCA AUACCCAAAAAU UAAA UUC AAAA AUAGAAUA \

UGA AGUA AGGU UGUGGGUUUUUA AUUU AAG UUUU UGUUUUAU U

UA - UU- AUAAGUA UUUUUUUU UAUA - UU

***Oryza sativa miRNA precursor:***

G GUC A AAA- .-AUUUUA CG - AAA

UGGGUGCGUACA GCC CAUCCAU CAC AAUC GC CAUG C A

AUCCACGUGUGU UGG GUGGGUA GUG UUAG UG GUAC G A

G A-- C CUAC \ ------ AU U GCU

A---------- UG

GUGC \

CACG G

AGAUCAACACC GA

**>miR164c UGGAGAAGCAGGGCACGUGCG**

***Arabidopsis thaliana miRNA precursor:***

U UA U A A C CACA AA CGG

GGG GAG ACAC UG UGGAG AG AGGGCACGUGCGAA AAUG AUCGAU U

CCC CUC UGUG AC ACCUC UC UCUUGUGCACGCUU UUAU UAGUUG A

- UC C A A A ---- AC UUC

***Oryza sativa miRNA precursor:***

C G - AUAU - AUCAUCUUCUUCCUCCUCCUCU - UG

UGGAGAAG AGG CACGUGCA UGC GUUC AUC AGCU CCAGCCU \

ACCUCUUC UCU GUGCACGU ACG CAAG UAG UUGA GGUUGGG U

C G C CU-- A AU-------------------- A UG

**>miR396b UUUCCACAGCUUUCUUGAACU**

***Arabidopsis thaliana miRNA precursor:***

A .-UUUUUCAUUUCCAUUG AAC

UUUUCCACAGCUUUCUUGA CUUUC UUUUUUUCUUA A

AAAGGGUGUCGAAAGAACU GAAGG AAAAGAAGAAU A

C \ ---------------- GAA

AAAA-- UAA

CUU \

GAA G

UUUUAC UUA

***Oryza sativa miRNA precursor:***

A - UG CU A A UCA----- GA GAU

CUUUCCACAGCUUUCUUGA CUUCU CUUG C C CUC CUU UUACUG GA A

GAAGGGUGUCGAAAGAACU GGAGA GAAC G G GAG GGA GGUGAC CU U

G A GU AG A - UAUUCGAA UA ACG

**>At5g36280_5_3 GUUUGCUUGUCGCUGGCGACU**

***Arabidopsis thaliana miRNA precursor:***

U U .-A ----- C A

CG UUGCU GUCGCUGGCGACUG AACCAGCGACAG CGA CAA \

GC AGCGA CAGCGACCGCUGAC UUGGUCGCUGUC GCU GUU A

C C \ - CGUUU U G

CA GACCA

GC \

UG A

AG AUCAA

***Oryza sativa miRNA precursor:***

-- U G C-- G-- - A UUCUUC U

GGU UCA GUUUGCUU U GCU GCGU CUGA UUG GUG C

CCA AGU CAGGCGAA G CGA UGCG GGCU AGC UAC U

AA - A AAU AUG C A UUUUAC G

**>At5g39200_5_10_rc UGAGUUGAGUUGAGUCGACUC**

***Arabidopsis thaliana miRNA precursor:***

C-- A--- -- .-U UCAA

UCAAA CA UUGACUCA UCAACUCA UUGACUCA C

AGUUU GU AGCUGAGU AGUUGAGU AACUGAGU U

UAA ACUC UG \ - UUAC

AA UUA A

CU AACUC U

GG UUGGG U

UG CAC A

***Oryza sativa miRNA precursor:***

A CC UA UAA UAGCA GA GG - AAU G UG

AUAU AG ACUU GCUUA UCAGCUCA ACC UUGAU GGACAAA CUG GU CAG \

UGUA UC UGAG UGAGU AGUUGAGU UGG GACUA CCUGUUU GAC CG GUC C

C -- C- UG- UUA-- AG A- A CUU - GA

**>At5g63700_5_1_rc UGACACAGGUCACUCAAACAG**

***Arabidopsis thaliana miRNA precursor:***

GA GGU - - - AA--- -- A CU

UUAG AAGUAGA UGGC GUUUGGGUGAU UG GUCA GC GA UC \

AAUC UUUAUUU AUCG CAAACUCACUG AC CAGU UG CU AG U

GG --- A G A CAUAA AU A AA

***Oryza sativa miRNA precursor:***

CA A AA--- UAG A GU UUA

CAAGUUA UGU UAAGUGA GUUA GU UU CA U

GUUCGAU ACA AUUCACU CAGU UA AA GU C

-- A GGACA UA- A UG UUA

**>miR393a UCCAAAGGGAUCGCAUUGAUC**

***Arabidopsis thaliana miRNA precursor:***

A C U U- .-A U A

GAGGA GGAUCCAAAGGGAU GCAU GAUCC AAUUA GG GA U

UUCCU CUUAGGUUUCUCUA CGUA CUAGG UUGGU CC CU U

A U - UU \ - C C

AUAUUUUCUUUAUAAUU AAAUC

GGCAAAU \

UCGUUUA A

----------------- AAAAC

***Oryza sativa miRNA precursor:***

C A - G .-AGCU G GA- AC

GGAAGC UCCAAA GGAUCGCAU GAUCC UCA GCUU UA CG \

CCUUCG AGGUUU CCUAGCGUA CUAGG AGU CGAA GU GC G

U C A A \ ---- G ACC CA

GA G

GC A

CG G

AG A

**>At1g25460_26 UUCACAAGUCACAAUCACCA**

***Arabidopsis thaliana miRNA precursor:***

- A A CC AGA U U CAUUU

GGU AGUGG GA UG ACUUG GAG CG CUA \

CCG UUACC CU AC UGAAC CUU GC GGU C

A A A AC A-- - U UUUUA

***Oryza sativa miRNA precursor:***

AC- GGC UG CCAAUCA U

UGGUGA UGAC UGAGAU GAG UCUUA \

ACCACU ACUG ACUUUA CUC AGAAU C

AAU AAC CA ACUCCC- A

**>At3g27883_1009 AGAUUCGAACUCUAGUCCUC**

***Arabidopsis thaliana miRNA precursor:***

CAAG ACA UAU GU UU

UGGAG UGGA UCGAA UCUU CAU \

AUCUC AUCU AGCUU AGAA GUG U

CUG- CA- --- AG UC

***Oryza sativa miRNA precursor:***

C C--- --- A G AA UUG

GAGG CUU UUGAGUCU CU GCAUG AAU AGAUGGG U

CUCC GAA AGCUUAGA GA CGUGC UUA UUUAUUU A

U CUCA AUU C G A- UUA

**>At5g02330_2413_1_rc CCGAACUCAUUUUGGUUUUUG**

***Arabidopsis thaliana miRNA precursor:***

C--- -- U UUU- AU UUAAA U

AUGACUUA ACC GAACUCAUUU GGUU GUU GUU UUGGG \

UAUUGAGU UGG UUUGAGUAAA CUAA CAA CAA AACCC A

AAAU UA - UAUU CU CUC-- A

***Oryza sativa miRNA precursor:***

AC-- UU- --- G C G

UCCGA ACAUU GGUUUUUCU UA GA CU U

AGGCU UGUGG UUAAAAGGA GU CU GA U

GCAC UUU AAA G C U

**>At5g32470_7631_1_rc GAGAAUAUGUGAGCUGGUAAA**

***Arabidopsis thaliana miRNA precursor:***

AUA - C - GAGUA

UUAGAGA UGUG AG UGGUAAAUUG GUUG \

AGUUUCU ACAC UU ACCGUUUAAU CGAU C

AAA A U U AUAAG

***Oryza sativa miRNA precursor:***

A A GA A---- U UU GGU

GUACUG GA UAGGU GCUGGU AAAUG CCA UCAGA \

CAUGAU CU GUCCG UGACUA UUUAC GGU GGUCU A

C - -- GAAGG U UU GUA

**>At2g28470_1616_rc UUUGUACUUUGUAGGUUUGG**

***Arabidopsis thaliana miRNA precursor:***

UU- U UAA AA ---------- UUA

GGUUUGUAC UGUAGGUU GG UAAUU ACGC AGAAA A

UCAAACAUG AUAUCUAA CC AUUAA UGUG UCUUU A

UAU - UAA AA GAUGAGGUGU UGU

.

***Oryza sativa miRNA precursor:***

U G- UU- GG C GA

UUUUG ACUUU CAGG U G GCUGUG U

AAAGU UGAAA GUUC G U CGACAC G

- AG UUU UU - AG

**>At1g03620_1175_1 UAGUGAUCUGAUUGGUUGAGG**

***Arabidopsis thaliana miRNA precursor:***

A GGU --- G- AA

CCU UCAAC GAUCAGAUCA GUU AGA--GUU \

GGG AGUUG UUAGUCUAGU CAA UCU CAA C

- G-- GAU AG \ AA

AAUAUUAAAGCC AAGGCCC

CAUCUC A

GUAGAG A

AAGAA------- AGCAAAU

***Oryza sativa miRNA precursor:***

UC G --- C- U U UUUUUGGUUUU UUAAU

GCUCA CC UC CGAUU GUCAUCGUA GCU CG GC \

CGGGU GG AG GUUAG UAGUGGUGU CGG GU UG U

GU G UUA UC - U UUUUGUUUUU- UUUUU

**>At1g11380_95 UUGGUCUUGUUCAGUUCUGUU**

***Arabidopsis thaliana miRNA precursor:***

A A - - AAUGUA UG

GAAUA AA UGAAUA GACUAGAA AC ACAA U

CUUGU UU ACUUGU CUGGUUUU UG UGUU A

C G U A GUG--- UU

***Oryza sativa miRNA precursor:***

CACAAA CAAU UUA C AGAUC U

UGCA GAU AAUAGA AGCAA GCGG GAA GAG U

ACGU CUA UUGUCU UUGUU UGUC CUU CUC C

C----- UGAC C-- U GU--- U

**>At1g13250_7364_1 CUCUAAUUGGCUCUUUGCAUA**

***Arabidopsis thaliana miRNA precursor:***

GGA GG AAA UC CUCA U

AUU GCA GAGAGCCAAU AG GAAU CGA A

UAA CGU UUCUCGGUUA UC CUUG GCU U

AUA -- A-- U- AC-- U

***Oryza sativa miRNA precursor:***

UUA- UG C UU A

AUAUGU CAGC G UUA GU C

UAUACG GUCG U AAU CG C

UUUC GU - CU U

**>miR169h UAGCCAAGGAUGACUUGCCUG**

***Arabidopsis thaliana miRNA precursor:***

G - U U - UUUUA UAUAUA A CAC G

UUGU UGG UAGCCAAGGA GACU GCCUG CG GACCA UC AAGACU UC A

AACA ACU AUCGGUUCCU CUGA CGGAC GC UUGGU AG UUCUGA AG U

A U - - U UGG-- UG---- A U-- C

***Oryza sativa miRNA precursor:***

U - U U GU- U GAU - U GAU

CUC GGU AGCCAAGGA GACU GCCUAUU GCUC UCUGAAU GC AG GCCAU \

GAG CCG UCGGUUCCU CUGA CGGAUAG CGAG AGACUUG CG UC CGGUG C

U A - - AGC U GU- G - UGA

**>miR399a UCUGCCAAAGGAGAUUUGCCC**

***Arabidopsis thaliana miRNA precursor:***

GAAAUGC A A A - C UCUU- CU

GGUG AUUACAGGGUA GAUCUCU UUGGCAGG AAC CAUUA UUAGA UGCAU C

CCAC UAAUGUCCCGU UUAGAGG AACCGUCU UUG GUGAU AAUUU ACGUA U

GUCUUCU - A A A U UCGUU UU

***Oryza sativa miRNA precursor:***

-- C UC UC A AU--- --- CUU

UAAGC AGUCCAGUUU AGGGC CUCUC UUGGCAGGG GC GUGA AGU U

AUUCG UCAGGUCAAA UCCCG GAGAG AACCGUCUC CG UACU UCG U

AC A UU GA C ACUUU CAC AUG

**>At1g32200_463_rc GUUGUCGACGUCACAUGAUUU**

***Arabidopsis thaliana miRNA precursor:***

UUC A U --- G

UA U CAU UAUGU U

AU A GUA AUACA A

AGA C - ACG C

C - C A A A AU A /- CAAAC

AUUUUU GUU GU G CGUC C UG UU UAUUU CUU A

UAAAAA CAA CA C GCAG G AU AA AUAAA GAA A

A U A - - C GU C CG - ACACA

***Oryza sativa miRNA precursor:***

.-UG - UC C UUGA

GGAA CUAAAUA GUUG GACGUCG AUGAUUU \

CCUU GAUUUAU UAAC UUGUAGU UACUAAA U

\ -- A U- - UCGA

**>At1g38122_654 UCAGAUGAAGAUGGAGAUGGA**

***Arabidopsis thaliana miRNA precursor:***

UC U - CA AG G ------- G

GU AAGU UUG AAU GAUGA AUGGAGAUG A UGUGAUU U

UA UUCA AAC UUA UUAUU UACCUCUAC U AUACUGG A

UC U G AG G- A UAAAACA U

***Oryza sativa miRNA precursor:***

A AA A A AU - - A AGGAA

GC GC GAG UGGAG UGGAG GG AGAU GGAUUG GG \

CG CG CUC GCCUC GCCUC CC UCUA CCUAAC CC G

C C- C C -- G A G GAAAA

**>At1g41790_1104_1 GCCAUCUCCUCGUCAUUGUGC**

***Arabidopsis thaliana miRNA precursor:***

- G UCCU - - .-CCAUA U U

UC AGUA AAUG GGAGAUG UAG UCAU UCUGUU GAG G

AG UCGU UUAC CCUCUAC GUC AGUG GGACGA CUC U

C G UGCU C U \ ----- - A

CA CG

CGAG \

GUUC G

UA UU

***Oryza sativa miRNA precursor:***

A-- - GU - GA CAC - U

GGCGAGGA GGAGCG GGUG GAGG GA GC CGG GG \

CCGCUCCU CCUCGU UCAC CUCC CU CG GCC CC C

CUG G UG U AC U-- G G

**>At1g42080_527 GAGUCUCAAUAGGAAUUUAGA**

***Arabidopsis thaliana miRNA precursor:***

UU C-- G AUU UU UGUGU

UC UCU UU CUAUU GAUUUCU--UAUUCAGAC UUAUU \

AG AGA AA GAUAA CUGAGGA AUGAGUCUG AAUAA U

UU UUU G CU- \ U- UUUCC

AUC U

ACCU A

UGGA U

AUU U

***Oryza sativa miRNA precursor:***

U CCCA AC .-CAUA AUCCUU AAGUG AA

GCUUGA UUGC GA CUU UCCUUG CCCAUUA CAGUG \

CGGAUU AAUG CU GAG GGGAAU GGGUGAU GUCAC G

U AUAA CU \ ---- GC---- A---- AU

CC U

UAUAGCC A

AUAUUGG A

A- U

**>At1g53360_1326_rc GCAGAGACUAUGUUUGGUUUG**

***Arabidopsis thaliana miRNA precursor:***

A - - AUUC- GUUGACUUC

GCAGAG CUAU GUUUGGU UUG UGAGC C

UGUCUC GAUA CAAACCG AAC AUUCG A

G G C GUUUU ACUUUUAGC

***Oryza sativa miRNA precursor:***

CA UGUU - AU U .-AA - AAUU

GGA GAGCCUA UG GUUUG ACCAGA UUU AAUA UGGCUA \

CCU CUUGGGU AC CAGAU UGGUUU AAA UUAU ACCGGU A

AA UUU- A -- U \ -- C ACGA

AAUUAUU C- CG

UAACUUAAAU UAAA U

AUUGAAUUUA AUUU A

AAACC-- UU AU

**>miR169d UGAGCCAAGGAUGACUUGCCG**

***Arabidopsis thaliana miRNA precursor:***

AUU U U A AUGU CA UU

GAAAA AAAGAA GAGAU GAGCCAAGG UGACUUGCCG UAUCAA AAUC A

CUUUU UUUCUU CUUUG CUCGGUUCC GUUGAACGGC GUGGUU UUAG A

CU- C U A CU-- -- UC

***Oryza sativa miRNA precursor:***

AC- A C - CCUGGUA G AAUC U

GAGGAGGAGA GGG UG AGCCAAGGAU GACUUGCCGGCU UU GGGG UCAGC \

CUUCUUCUCU UCC AC UCGGUUCCUG CUGAACGGCCGA AG UUCC AGUCG U

CGA - A U UUG---- G GCGA U

**>At1g61840_4217 CCAUGACUCUCUUUUUCUUCU**

***Arabidopsis thaliana miRNA precursor:***

U --- CUCCUCA

GAGAA GAAA AGGGUCAUGGUU C

UUCUU CUUU UCUCAGUACCGG U

- UUC UACUACG

***Oryza sativa miRNA precursor:***

U--- UU U U A ---- AUUGAU- U G G

AUGG UGG GAUGG GG GU GUGGA GGAUA GGC A UUGAU A

UACC ACU UUAUU UC CA UACCU CUUAU UCG U GAUUA U

UUCU UC C U G UAUU CUUUGUU - G A

**>At1g66810_329_1 GGUUUUGUUUGUAACUUGGAG**

***Arabidopsis thaliana miRNA precursor:***

-- AAG A U .-AAGCAAAA GAA

UUC AUU CAAGU UAUAAAUA GAACCUC AUCUAUUUU \

AGG UAA GUUCA AUGUUUGU UUUGGAG UAGAUAAAA A

AA GAG - - \ -------- AAA

GGA---- A

AACAAA \

UUGUUU A

GGGAGGA G

***Oryza sativa miRNA precursor:***

AACG -UG - A UAAU -- .-AUAUA U

CAUU CCA UA AGACAA GACC CAAAUGA UCC GGCG \

GUAG GGU AU UUUGUU UUGG GUUUACU AGG CUGC C

GA-- UGA G - CAC- CC \ ----- A

AGUUAAAAC -- A

CGG GUC A

GCC CAG A

A-------- GU A

**>miR414 UCAUCUUCAUCAUCAUCGUCA**

***Arabidopsis thaliana miRNA precursor:***

A UUA UC A A--- A - A G CAUCAUCA

UC UCA UCA AUC UCAUAUUC UCUUC UC AUC UC UCAU U

AG AGU AGU UAG AGUAUGAG AGAAG AG UAG AG AGUA C

- UG- U- - AGUG - A A - UGCUACUA

***Oryza sativa miRNA precursor:***

G G - A- A AU A G GCAUGAAA G A- A

GUUGCC CU CCG UC UCCUC UC C UC UCCUU CC CC GCAA U

CGGCGG GA GGC GG AGGGG AG G AG GGGAG GG GG CGUU C

G - U GG G GG A G -------- G CG C

**>At1g73320_140_rc UGCUUGAGAGUUGAGACAA**

***Arabidopsis thaliana miRNA precursor:***

----- A C GC -- U UGU CA UU AAA

AUUGUUA UGCUUGAG GUUGAGA AAU CA AG UUGUGA AAC ACGGU CUAUG U

UAACAAU AUGAAUUC CAAUUCU UUA GU UC AAUAUU UUG UGUUA GAUGC A

CAACA A A A- AA U --- UG UU AAA

***Oryza sativa miRNA precursor:***

U GAG GA .-A GAAAA - C UCUGCAC A UU

UAUGC UGA UUGA CAA CACGAAA GUGU CAUU GUUU GC CAUUU \

AUACG ACU AAUU GUU GUGUUUU UACA GUAA CAAA UG GUAAG U

U AAA UC \ - ----- U - U------ - UU

AAAA U

AUUC A

UAAG U

GA-- A

**>At1g75680_601 GGUGGUUCAUUGUUGUUGCCA**

***Arabidopsis thaliana miRNA precursor:***

G --- - A AAGAAGG

AUGG AACAACAA ACU UUAC CGUU \

UACC UUGUUGUU UGG GGUG GCGG A

G ACU U A GUUAACA

***Oryza sativa miRNA precursor:***

UACU -- - UU C---- ----- U- U --- AA UUGA

UCUC GC CAA AAUG CAGAC UGGGU CACUU AC GAC CAGAG UUC \

AGAG CG GUU UUAC GUUUG ACUCG GUGAG UG CUG GUCUU AAG U

UUAC UU G UU UCUUA AAAAU CU U AUU GG UCUU

**>At3g08760_3637_rc ACAUGCAGAUUUUGCAUUUG**

***Arabidopsis thaliana miRNA precursor:***

C- U CA - C C - UU- AG

UUGA AUGCAGAUU UG UUUGU AG UU GU UC AG \

AACU UACGUCUAA AC AAACA UC AA CA GG UC U

UU C AC A C U C UAU UU

***Oryza sativa miRNA precursor:***

GAAC AUUUU UU -- A .-CUACAACAAA A- GGCAC

UUAU AUUCAG GCAU GAUUCU GUAG GAA UAGAU GAU \

GAUA UAAGUC UGUA CUAAGA CAUC CUU AUUUA CUA C

AUC- GAUAU CC UU A \ ---------- GG AAAAU

AAUAU AAAUA

GC \

CG U

AUU-- ACCAA

**>miR418 UAAUGUGAUGAUGAACUGACC**

***Arabidopsis thaliana miRNA precursor:***

AU A-- UA- AAAA AAA GA

UUUAA UUAG AUC GCGU AGA UCC A

AGAUU AGUC UAG UGUA UCU AGG U

CC AAG UAG A--- CA- AC

***Oryza sativa miRNA precursor:***

-- C CAU

UGC CAUU UUAUCAUCGCA \

ACG GUAA AGUAGUAGUGU U

CA - AAU

**>At4g11130_6601_rc AGUGAUGGCCAUGGCAUGGAA**

***Arabidopsis thaliana miRNA precursor:***

AUUAUG GGC AU A - -- AAUCA

GAUA AGUGAU CAUGGC GGAAGAAGGUG GACUUGCA UGA AUCGUG A

UUAU UCACUA GUACCG CCUUUUUCCAU CUGAACGU ACU UAGUAC A

GAG--- AUA AU G C AU CUCCA

***Oryza sativa miRNA precursor:***

AUU GA - UUGC- G .-AACA - AAAA

UCUCG AGU UGG CCA AUGGA UG GAU GGGACU C

AGAGC UUA AUC GGU UACCU AC CUA CCCUGA U

GU- -- A UCUAA - \ ---- U AUUU

ACA----------- GG AAAUUAA

UC AUGUUUGA U

AG UGCAAAUU U

CCAAAAUAAUUAUC A- CUAAAUA

**>At4g12190_99_rc UCUCUCUGUUUUGCAUUAUCA**

***Arabidopsis thaliana miRNA precursor:***

-- CAUUA A AA C

GUCUCU CUGUUUUG UC GUGA UUA \

CAGAGA GACAAAAC AG CACU AAU A

AG A---- A GC G

***Oryza sativa miRNA precursor:***

AA - CU UCC A G .-UAU C

GAAUUG UU CU CUGUUU AUU UC UAUA GCAGU A

CUUGAU GA GA GAUAGA UAA AG AUAU CGUCG A

-- U AG UC- A A \ --- U

C UUAUG AA

UCC CUC \

AGG GAG U

U UA--- UA

**>At4g13550_1386 UCAACGAUGCACUCAAUGAUG**

***Arabidopsis thaliana miRNA precursor:***

A- A A A C A UACUU- UAA A A

GAA AACAU AUUGA UGCA CG UGAU UA CUU UUA UUCA C

CUU UUGUA UAACU ACGU GC ACUA AU GAA AAU AGGU A

AC G C A A C UAAAAU UAA A A

***Oryza sativa miRNA precursor:***

UGC C G A C UGAUCAGAUC - AA CGC

GGCAGAGA CAAA GCAU AUUGAGUG AGCG UGAUGA ACCGG CCA UG A

UUGUCUCU GUUU CGUA UAACUCAC UCGC ACUACU UGGCC GGU AC U

UA- A G G A UGUUGCCAU- A -- AAC

**>At4g16100_467 UAUGCGUGUUAUGGAUAUG**

***Arabidopsis thaliana miRNA precursor:***

- AUG- U ----- C AG C

UCUAUAU AUAU AAUACGC AUACG UCUA GGAUU AC \

AGAUAUG UAUA UUGUGCG UAUGU AGAU UUUAA UG A

G GGUA - UUUCA - AA A

***Oryza sativa miRNA precursor:***

GGA- A A- AG

CCA CA AACAA AUAUACU U

GGU GU UUGUU UAUAUGA U

AUAG A CG CA

**>At4g29200_13 UCACAACUCUCAAGGUUUUGU**

***Arabidopsis thaliana miRNA precursor:***

CAAC - A A CAA A UCUCUCGUCACAACU

ACGAAACCU GA AG UGUGA CG ACU AC C

UGUUUUGGA CU UC ACACU GC UGA UG U

A--- C A - --- C CUCUCUGACUCAAAU

***Oryza sativa miRNA precursor:***

A C A ---- C

UUC CACA AAAUCUU AG GUGAUC G

AGG GUGU UUUGGAA UC CACUAG A

- - C UAAA U

**>At4g38520_371_1 GGACGAGAGGGAAGUGGAGCC**

***Arabidopsis thaliana miRNA precursor:***

GA - - - A - CGAA UGGGACC

AAAAG CGA GA GG GA GU GGAGCC AGC \

UUUUC GUU CU CC CU CA CCUCGG UCG U

UG G A G C G ---- UGUAGUU

***Oryza sativa miRNA precursor:***

G C U C UG U - GG UC------ UCG

GGUUU GA GUGAGGGAAG GGAG GG GA GGAG G GACU ACGC \

UCGGG CU UACUUUUUUU UCUC CC CU UCUC C UUGG UGCG U

G - - U CU C U UU UUCACUUU UGC

**>miR172b* GCAGCACCAUUAAGAUUCAC**

***Arabidopsis thaliana miRNA precursor:***

U A GC C A GAA UGA U A

GUCGUUG UUGU G GCAGCA CAUUAAGAUUC CAUG AU UAAA ACCCUA \

CGGCAGC AACA C CGUCGU GUAGUUCUAAG GUAU UA GUUU UGGGAU A

U A UA A A AUG UA- - U

***Oryza sativa miRNA precursor:***

- C A C A ----- U U CUAAA A

AGUCG GUG UUGC GGUGCAGCA CAUCAAGAUUC CAUCG AGU CA CC CG G

UCAGC CGC AACG CUACGUCGU GUAGUUCUAAG GUAGU UCA GU GG GC A

U A A A A GUAUA - C UUGGA U

**>miR398b UGUGUUCUCAGGUCACCCCUG**

***Arabidopsis thaliana miRNA precursor:***

U A U A C U CAAC --- C

GGA CUCG CAGGG UGAU UGAGAACACA GAG AAU GGCUGUA AUGACG \

UCU GAGU GUCCC ACUG ACUCUUGUGU CUU UUG UCGACAU UACUGC U

C C C G A - CUC- UGU A

***Oryza sativa miRNA precursor:***

CU A GUA G .-CAAUACAA A

GAGAAG GAACCCAGAGG GUG CUGAGAACACAG UGC UGUAUGGUG \

CUCUUC UUUGGGUUUCC CAC GACUCUUGUGUC AUG AUAUGUCAU G

-- C UG- A \ -------- C

AU G

GGA U

CUU A

U- A

**>At5g20490_133 GAGAGACCGAUUUUGCAGAAA**

***Arabidopsis thaliana miRNA precursor:***

CAAU C- UUUCCC U

UUUC AGAUC UCUCUCUU AG U

AAAG UUUAG AGAGAGAA UC A

ACGU CC CCUAC- A

***Oryza sativa miRNA precursor:***

C U U C G C

UCUUUU UCU UAGA UC GAC UUC \

AGAAGA AGA GUUU AG CUG GAG C

- C U C A G

**>At5g25610_3237 CACUCCAAAUCUCCAACUCGU**

***Arabidopsis thaliana miRNA precursor:***

AAUUA C .-UAAUA UG

UUACGAGU GAGA UUGGAGUGAUGC GUUUCU \

AAUGCUCA CUCU AACCUCACUACG UAGAGG A

AC--- A \ ----- CU

GGAUG AAA

GAUA \

CUAU A

A---- AAA

***Oryza sativa miRNA precursor:***

- AA U UC AU C - CG G

UAUUU UGAU CGACG AAUU GA AUUUGGA UGC--ACUUG UC CC G

GUAGA GCUG GCUGC UUAA CU UAAACCU ACG UGAAC AG GG G

C CC - C- C- C \ G AG A

GGU U --- G

CG GGGGGUCGUG GCG A

GC CCCCCGGUGC UGC C

U-- - AGG U

**>At5g38490_130_1 AAUGCAGAAAACAGUGGAGUC**

***Arabidopsis thaliana miRNA precursor:***

------------- -- GAUG UUA UUUUG- A

UUUG CUUCACU UUUUUGUAUUU UA UUAUAUU GGC C

AGAC GAGGUGA AAAGACGUAAA AU AAUAUAG CCG U

UUAAAAAAACUCU CA AUAA UA- UUUCAG U

***Oryza sativa miRNA precursor:***

UUA AA- AA- A A C U CU

GUG GUUAG CACUGU UUG CA UC UGCC GCUCAUUA \

UAC CAAUU GUGACA GAC GU AG AUGG UGGGUAGU U

UC- GAG AAA - A A - AA

**>At5g38550_198_rc CAAAUGAUCCCUUGUCUC**

***Arabidopsis thaliana miRNA precursor:***

AAAA AUCC UC AA A UG CAA C

UGA CAAAUG CUUG UCA UGGA CU A AACA A

ACU GUUUAC GAAC AGU GCUU GA U UUGU C

A--- ACGA C- A- A GU AGG A

***Oryza sativa miRNA precursor:***

UU- AA AU UU- .-CU - AAG--- ACAC

GGGCA CAAA ACAA GAUCUC GUCUCU UG AUCCU GU \

CCUGU GUUU UGUU CUAGAG UAGAGA AC UGGGA CA A

CUU A- CC CCC \ -- A GGAAAA CAGU

UUU--- GG UUG

G GGC \

C UCG C

AUCUCU UU UAA

**>At5g40770_3770_rc GAAUUGUGAAUUUGUGAUCGU**

***Arabidopsis thaliana miRNA precursor:***

- - G UG- G C CUAACCCAA

GGU UUUG U GAAU UGAAUUU UGAU GUAU A

CCA AAAC A CUUA ACUUAAA AUUA CAUA U

U U G UAA - - UACACUUUC

***Oryza sativa miRNA precursor:***

AC AU U- CU A

GAGUGGG GA UGUGAAU UGUGAU CAGC G

CUUACCU CU AUACUUG ACGCUA GUUG U

AC -- UC CU A

**>At5g55830_309_rc UUGACAGAAGAAAGAGAGCAC**

***Arabidopsis thaliana miRNA precursor:***

AAAA U A - A- GAUUA AG

GAUGA UG UG CAGAAG AAAGAGAGCACA CCUGG GCAAAA A

UUACU AC AC GUCUUC UUUCUCUCGUGU GGGCU CGUUUU U

---- C C C GA GUUCC GA

***Oryza sativa miRNA precursor:***

TG - G- A-- - C GAC

G AUGACAGA AGA AGAGAGCACA CCC GGCAG AGCGAC \

C UACUGUCU UCU UCUCUCGUGU GGG CCGUC UCGCUG G

GU A AG GCC A U GCG

**>At5g56680_271 UUCUCUAUCGAUCUCCAUUGU**

***Arabidopsis thaliana miRNA precursor:***

A A U C C GCAAGU UC UG

ACGAUG AGA UG UA GAGAA AA GA UCAGC A

UGUUAC UCU GC AU CUCUU UU CU AGUUG U

C A U - - AACAU- UA CU

***Oryza sativa miRNA precursor:***

UA-- U U GA .-GCAACAAAACAAUUCA CC

GUGGC GAG UC GG AGAAG ACAAG U

CAUCG CUC AG UC UCUUC UGUUC U

UUAC U C UC \ ---------------- UU

CAGA UG U

GGG GU C A

CCC UA G C

CACC GU A

AU U G

GGCA GCGC C

UCGU CGCG G

G- C C

**>At5g57630_760 UGUUGCUUCCUGUUUAUG**

***Arabidopsis thaliana miRNA precursor:***

C C -- UAC UUA .-UU AA

UGAA CAU AAC GAAGCAACAA GAA GC GCUUAAG U

AUUU GUA UUG CUUCGUUGUU CUU CG CGAGUUU U

C U UC U-- UCA \ -- AA

UAUUCCAUUU UU

UC \

AG U

UUUC------ AC

***Oryza sativa miRNA precursor:***

UU ACA - CA U UAA

UUACC GACUA GC UGAAU GAA CACCG \

AGUGG UUGGU UG AUUUG CUU GUGGU A

U- GG- U UC C UUG

**>miR172e GAAUCUUGAUGAUGCUGCAUC**

***Arabidopsis thaliana miRNA precursor:***

GUC A C A AGA U UU --- UU- CC

GUA GC GAUGCAGCA CAUUAAGAUUC CA GAUG GG CCCUU UGC UCG \

CAU CG CUACGUCGU GUAGUUCUAAG GU CUAU CC GGGAA ACG AGC U

AAA A A G GAG U UU AAG CCU UC

***Oryza sativa miRNA precursor:***

- C A C A ----- U U CUAAA A

AGUCG GUG UUGC GGUGCAGCA CAUCAAGAUUC CAUCG AGU CA CC CG G

UCAGC CGC AACG CUACGUCGU GUAGUUCUAAG GUAGU UCA GU GG GC A

U A A A A GUAUA - C UUGGA U

**>At5g62160_613_rc GGGCAACUCUCCUUUGGCAAG**

***Arabidopsis thaliana miRNA precursor:***

C A C CU A AUA CAAUA-- AAGAA

GCAGUGA AGGGCA CU UC UUGGCA GUGAC GC GUC C

CGUCAUU UCCCGU GA AG AACCGU CGCUG CG UAG A

A A A AU C AAC AUAAACA AAAAC

***Oryza sativa miRNA precursor:***

UG AA C A UGAAAAUGA - - AC

UAAGC AGUCCAGUUUUAGGGC CUCUC UUUGGCAG GGGC GUGA G CU \

AUUCG UCAGGUCAAAGUCCCG GAGAG GAACCGUC CUCG CACU C GA A

-- AG A C UA------- U A AA

**>miR420 UAAACUAAUCACGGAAAUGCA**

***Arabidopsis thaliana miRNA precursor:***

AAUCAC-- G AAAAU UACU CUAUCAAC

CUAAACU GGAAAU CA UGGA ACA U

GAUUUGA CCUUUA GU AUUU UGU U

GUAAUUUA A GCUCU CUCU UUUCUUUU

***Oryza sativa miRNA precursor:***

CGG A UUG--- -- AAA CCAA CU A A C A

UAAAUUAAUCA AAAUG UCU AAU UGUUAA UACUU UG CAA UA UU CAAGA A

AUUUAAUUAGU UUUAC AGA UUA ACAAUU AUGAA AC GUU AU AA GUUCU A

AA- C UUAAUA AA C-- ACUC AG C A A A

**>miR398a UGUGUUCUCAGGUCACCCCUU**

***Arabidopsis thaliana miRNA precursor:***

A A U UA-- CU UUCAAA

UUCAAAGG GUGGC UG GAACACA UC AUGGUUUC U

AAGUUUCC CACUG AC CUUGUGU AG UACCAAAG U

C G U UUUG U- UUACCU

**Oryza sativa miRNA precursor:**

CU A GUA G .-CAAUACAA A

GAGAAG GAACCCAGAGG GUG CUGAGAACACAG UGC UGUAUGGUG \

CUCUUC UUUGGGUUUCC CAC GACUCUUGUGUC AUG AUAUGUCAU G

-- C UG- A \ -------- C

AU G

GGA U

CUU A

U- A

**>miR416 GGUUCGUACGUACACUGUUCA**

***Arabidopsis thaliana miRNA precursor:***

AAC CC UU GCG- G- A

CGA UGAAC GGU GUACGUAUGGACC UC UUGG \

GCU ACUUG UCA CAUGCAUGCUUGG AG AACC A

--- -- -- ACCA AA U

***Oryza sativa miRNA precursor:***

G ------ GAA U -- UAC GG G A A

GAUGAG AGU AUGAAUA AGAA UAGA AAAGC GGU UU UUG GC \

CUACUU UCA UGCUUGU UCUU GUCU UUUCG UCA AA AAC CG G

G CAUGCC G-- U CG UC- AA A - C

**>miR172d AGAAUCUUGAUGAUGCUGCAG**

***Arabidopsis thaliana miRNA precursor:***

UA A U AG UUCUCU U UC

UUGC UUGCA CAUC UCAAGAUUC AAAUCAGA UA GGGUUU U

AACG GACGU GUAG AGUUCUAAG UUUGGUUU AU UCCGAG U

GC C U AG UUU--- U UU

***Oryza sativa miRNA precursor:***

A C A ----- U U CUAAA A

UUGC GGUGCAGCA CAUCAAGAUUC CAUCG AGU CA CC CG G

AACG CUACGUCGU GUAGUUCUAAG GUAGU UCA GU GG GC A

A A A GUAUA - C UUGGA U
