# Supplementary material for: Prediction and identification of Arabidopsis thaliana microRNAs and their mRNA targets
Source: Genome Biol. 2004 Aug 31;5(9):R65. doi: 10.1186/gb-2004-5-9-r65 (PMC522872; doi:10.1186/gb-2004-5-9-r65)
Supplement: Additional data file 3 — MPSS evidence for known and predicted Arabidopsis miRNAs [file gb-2004-5-9-r65-s3.doc]

**Additional data file 3. MPSS evidence for known and predicted *Arabidopsis* miRNAs.**

miR name miR sequence miR position No. of matching relationship to miRNAs

MPSS sequences

miR156b UGACAGAAGAGAGUGAGCAC Chr4, us of At4g30970(a) 2 242-nt us, 279-nt ds

miR156d UGACAGAAGAGAGUGAGCAC Chr5, us of At5g10940(s) 1 226-nt us

miR156f UGACAGAAGAGAGUGAGCAC Chr5, ds of At5g26150(a) 1 408-nt ds

miR157a UUGACAGAAGAUAGAGAGCAC Chr1, ds of At1g66780(a) 2 123-nt us, 336-nt ds

miR157c UUGACAGAAGAUAGAGAGCAC Chr3, ds of At3g18215(a) 1 364-nt ds

miR158 UCCCAAAUGUAGACAAAGCA Chr3, ds of At3g10750(s) 1 184-nt ds

miR159a UUUGGAUUGAAGGGAGCUCUA Chr1, ds of At1g73690(s) 2 249-nt ds, 252-nt ds

miR160a UGCCUGGCUCCCUGUAUGCCA Chr2, ds of At2g39180(a) 1 230-nt us

miR160c UGCCUGGCUCCCUGUAUGCCA Chr5, us of At5g46840(a) 1 71-nt us

miR162a UCGAUAAACCUCUGCAUCCAG Chr5, us of At5g08180(s) 4 15-nt overlap, 28-nt us,

183-nt us, 196-nt us

miR162b UCGAUAAACCUCUGCAUCCAG Chr5, us of At5g23060(s) 1 15-nt overlap

miR163 UUGAAGAGGACUUGGAACUUCGAU Chr1, ds of At1g66720(s) 1 372-nt us

miR164 UGGAGAAGCAGGGCACGUGCA Chr5, us of At5g01740(s) 1 481-nt ds

miR165a UCGGACCAGGCUUCAUCCCCC Chr1, ds of At1g01180(a) 1 155-nt ds

miR166a UCGGACCAGGCUUCAUUCCCC Chr2, us of At2g46690(a) 3 45-nt ds, 89-nt ds

360-nt ds

miR166b UCGGACCAGGCUUCAUUCCCC Chr3, us of At3g61900(a) 3 49-nt us, 171-nt ds

388-nt ds

miR167a UGAAGCUGCCAGCAUGAUCUA Chr3, us of At3g22890(a) 1 25-nt ds

miR168a UCGCUUGGUGCAGGUCGGGAA Chr4, us of At4g19390(a) 1 48-nt ds, 480-nt ds

miR171a UGAUUGAGCCGCGCCAAUAUC Chr3, ds of At3g51370(a) 1 68-nt us

miR172a AGAAUCUUGAUGAUGCUGCAU Chr5, ds of At5g04270(s) 1 418-nt ds

miR171b CGAUUGAGCCGUGCCAAUAUC Chr1, ds of At1g11730(a) 1 156-nt ds

miR413 AUAGUUUCUCUUGUUCUGCAC Chr1, ds of At1g62350(s) 1 321-nt ds

miR414 UCAUCUUCAUCAUCAUCGUCA Chr1, ds of At1g67190(a) 2 50-nt ds, 63-nt ds

miR415 AACAGAGCAGAAACAGAACAU Chr1, ds of At1g74450(s) 3 102-nt ds, 410-nt ds

487-nt ds

miR418 UAAUGUGAUGAUGAACUGACC Chr3, ds of At3g18890(a) 1 447-nt us

miR419 UUAUGAAUGCUGAGGAUGUUG Chr4, ds of At4g32440(s) 1 230-nt us

miR420 UAAACUAAUCACGGAAAUGCA Chr5, ds of At5g62840(s) 1 313-nt us

At4g31350_5_17 UUGAGACUUGAGACUGAACAU Chr4, ds of At4g31350(s) 3 22-nt ds, 162-nt us,

205-nt us

At1g11380_95 UUGGUCUUGUUCAGUUCUGUU Chr1, ds of At1g11380(a) 1 81-nt us

miR172b* GCAGCACCAUUAAGAUUCAC Chr5, ds of At5g04270(s) 1 439-nt ds

miR398b UGUGUUCUCAGGUCACCCCUG Chr5, ds of At5g14540(s) 1 83-nt us

At5g20490_133 GAGAGACCGAUUUUGCAGAAA Chr5, ds of At5g20490(s) 1 207-nt ds

miR171c UUGAGCCGUGCCAAUAUCACG Chr1, ds of At1g62030(s) 1 146-nt ds

miR390a AGCUCAGGAGGGAUAGCGCCA Chr2, ds of At2g38320(a) 1 242-nt us

At3g61890_5_33 UUGAGGGGACUGUUGUCUGG Chr3, ds of At3g61890(a) 3 49-nt ds, 286-nt ds

482-nt ds

At4g10845_5_11 GCAUUCAUGCAUACAUCCAU Chr4, ds of At4g10845(s) 1 488-nt ds

The distance between the genomic loci of a miRNA sequence and a MPSS match is shown as “relationship to miRNAs”.

Chr, chromosomal location of known *Arabidopsis* miRNAs; us, upstream; ds, downstream; a, antisense to nearest annotated gene; s, sense to nearest annotated gene.
